# The Mechanisms of Resistance to JAK Inhibitors in Lymphoid Leukemias: A Scoping Review of Evidence from Preclinical Models and Case Reports

**DOI:** 10.3390/ijms26189111

**Published:** 2025-09-18

**Authors:** Daniel Martínez Anaya, Marian Valladares Coyotecatl, Maria del Pilar Navarrete Meneses, Sergio Enríquez Flores, Patricia Pérez-Vera

**Affiliations:** 1 Laboratorio de Genética y Cáncer, Instituto Nacional de Pediatría, Mexico City 04530, Mexico; alejandro.bqd@gmail.com (D.M.A.); mazatl_coyotecatl@ciencias.unam.mx (M.V.C.); peachnavarrete@hotmail.com (M.d.P.N.M.); 2 Posgrado en Ciencias Biológicas, Universidad Nacional Autónoma de México, Mexico City 04510, Mexico; 3 Laboratorio de Biomoléculas y Salud Infantil, Instituto Nacional de Pediatría, Secretaría de Salud, Mexico City 04530, Mexico; sergioenriquez@ciencias.unam.mx

**Keywords:** lymphoid leukemias, JAK inhibitor, ruxolitinib, resistance mutation, molecular resistance, functional resistance

## Abstract

The use of JAK inhibitors (JAKi) represents a promising therapeutic approach for patients with lymphoid leukemias (Lym-L). Clinical trials are ongoing to evaluate the safety and efficacy of JAK inhibitors. Over the last years, there have been reports of preclinical Lym-L models that developed JAKi resistance, and reports of patients treated with JAKi who experienced treatment failure. Although evidence shows that there are diverse JAKi mechanisms, no review studies have been performed that summarize and discuss this information. This scoping review aimed to provide an updated overview of the mechanisms underlying JAKi molecular resistance in Lym-L. According to a scoping review PRISMA guidelines, a search was conducted in the PubMed and Europe PMC databases for studies published from 2010 to 2024. We included articles that described the molecular resistance to JAKi in Lym-L preclinical models or patients. The search was complemented by a review of laboratory-engineered resistant mutations in genomic datasets to obtain more information about their presence in patients with Lym-L. Twenty-two articles were eligible for this review, and six different mechanisms of molecular resistance were identified: (1) point mutations in the kinase domain, (2) cooperation between double-JAK mutants, (3) inactivation of phosphatases, (4) evasion of JAK inhibition due to trans-phosphorylation of JAK family proteins, (5) upregulation of pro-survival proteins, and (6) activation of kinase cross-signaling pathways. The integrated evidence enabled the identification of specific mechanisms of molecular resistance to JAKi in Lym-L, as well as promising therapeutic approaches to prevent them. These include selecting a sensitive JAKi, choosing an effective dosage regimen, and combining inhibitory molecules.

## 1. Introduction

The Janus kinases (JAKs) are a family of four non-receptor tyrosine kinases (JAK1, JAK2, JAK3, and TYK2) that phosphorylate cytokine receptors, creating docking sites for the activation of transcription proteins (STATs), which activate gene expression programs associated with differentiation, proliferation, and survival processes in hematopoietic progenitor cells [1,2]. Deregulation of JAK/STAT signaling is a hallmark of hematological malignancies, predominantly in myeloproliferative neoplasms (MPNs). However, precursor and mature lymphoid leukemias (Lym-L), including acute lymphoblastic leukemia (ALL) and T-cell prolymphocytic leukemia (T-PLL), also exhibit abnormalities that lead to JAK/STAT signaling malfunction [3,4,5]. ALL is the most common childhood neoplasm and results from the transformation of B-cell precursors (B-ALL), comprising 80% of cases. The remainder involves T-cell precursors (T-ALL) [6]. In contrast, T-PLL is an aggressive neoplasm of mature T lymphocytes that occurs in adulthood [4,7].

The four members of the JAK family are involved in Lym-L pathogenesis, and patients with *JAK* abnormalities experience poor outcomes and a high risk of relapse [8]. Activating mutations in *JAK1* and *JAK3* are predominantly found in T-ALL (6.5–27% and 10–16%, respectively), and most frequently in T-PLL (8–12.5% and 30–42%, respectively) [3,4]. Conversely, *JAK2* mutations are primarily observed in high-risk B-ALL (8.5%) and B-ALL patients with Down syndrome (18–28%). In addition, the JAK2 p.R683G/S activating mutation is present in half of Ph-like B-ALL patients with *CRLF2* rearrangements. Additionally, *JAK2* gene fusions resulting from rare chromosomal rearrangements are another activating alteration that occurs in approximately 7–14% of B-ALL Ph-like patients. Activating mutations in *TYK2* are present in about 21% of T-ALL patients. Furthermore, some rare activating gene fusions have been reported as part of the genomic landscape of the B-ALL Ph-like subtype [3,8,9,10].

In patients with MPNs, the predominance of hyperactive JAK2 signaling associated with the JAK2 p.V617F activating mutation (50–98%) has led to the development of JAK inhibitors (JAKis), which substantially alleviate the symptom burden [11,12]. There are two general categories of competitive JAKis: (1) type I inhibitors, which occupy the ATP-binding pocket in the active conformation (DFG-in), and (2) type II inhibitors, which additionally bind to the allosteric pocket available in the inactive conformation (DFG-out) [1,13,14]. Based on its effectiveness in MPNs, JAKis represent a promising approach for the treatment of JAK-mutated Lym-L. Ruxolitinib, a type I JAK1/2 inhibitor, is a candidate drug that has been approved by the US Food and Drug Administration (FDA) for treating MPNs [8,15]. The effectiveness of ruxolitinib against JAK-mutated Lym-L has been demonstrated in preclinical models and case reports [8,15,16,17,18].

Ruxolitinib significantly reduced circulating blast counts in murine xenograft models of B-ALL and T-ALL with *JAK* genomic lesions. Moreover, case reports of B-ALL patients with *JAK2* gene fusions who received ruxolitinib as part of their chemotherapy regimen have described long-term remission with undetectable minimal residual disease levels [16,19,20]. Additionally, in a preliminary study, Herbaux et al. (2021) reported that adding venetoclax (a BCL-2 inhibitor) to ruxolitinib treatment improved the clinical response in patients with JAK-mutated T-PLL who were refractory to conventional chemotherapy regimens [17,21]. Besides ruxolitinib, preclinical studies have shown that other type I JAKis approved by the FDA, such as fedratinib and tofacitinib, as well as type II JAKis BBT594 and CHZ868, exhibit striking efficacy in JAK-mutated ALL [14,22,23,24,25,26]. Currently, phase II clinical trials are underway to determine the safety and efficacy of ruxolitinib as a monotherapy or in combination with standard chemotherapeutic drugs in patients with high-risk B-ALL and T-ALL (clinical trial IDs https://clinicaltrials.gov. NCT03117751, NCT03571321, and NCT02723994), as well as in patients with peripheral T-cell leukemia/lymphoma diseases, including T-PLL (NCT02974647) [25].

Despite evidence supporting the progress of ruxolitinib through ongoing clinical trials, controversy remains due to differing preclinical studies and case reports that demonstrate reduced activity of ruxolitinib and other JAKis against JAK-mutated human lymphoid cells [16,24,26,27,28]. The phase I/II clinical trial NCT02420717, which aimed to investigate the combination of ruxolitinib with conventional chemotherapy in patients with relapsed or refractory Ph-like ALL, was discontinued before the phase I dose escalation due to the lack of response. Additionally, the phase II clinical trial NCT02974647 reported no observable clinical benefit in approximately 50% of patients with JAK/STAT-deregulated peripheral T-cell leukemia/lymphoma treated with ruxolitinib monotherapy [26]. Some reports have described the in vitro generation of mutations located near the ATP-binding site of the four JAK family members. These mutations decreased the potency of JAKis in ALL artificial systems. However, the clinical relevance of these laboratory-engineered mutations remains to be determined as experience regarding response to ruxolitinib in ALL patients is limited [8,15,22,24,28,29,30].

## 2. Rationale for the Scoping Review and Main Objective

Because JAKis have emerged as a promising therapeutic option for JAK-deregulated Lym-L and given the unsettling possibility of the emergence of clinical resistance in JAKi-treated patients, examining the available literature on JAKis resistance helps analyze strategies to avert this possible outcome. Although vast evidence shows that there are diverse JAKi mechanisms, to our knowledge, no review studies have been performed that summarize and discuss this information in Lym-L. The main objective of this scoping review is to gain a deeper understanding of the mechanisms underlying JAKis molecular resistance in Lym-L. We update and summarize the evidence published in the last decade and early 2020s. Unlike other reviews on this topic, we included the four JAK family members and focused on models that recapitulate the genetic rearrangements of Lym-L. In addition, we expanded existing knowledge by reviewing laboratory-engineered resistant mutations in previous genomic database annotations to learn more about their presence and possible clinical significance in patients with Lym-L. This scoping review integrating the evidence on the different biological mechanisms for JAKi would be helpful to motivate the search for ways to prevent JAKi resistance in the therapeutic field.

## 3. Materials and Methods

### 3.1. Information Sources and Search Strategy

Following the Preferred Reporting Items for Systematic Reviews and Meta-analyses extension for scoping reviews (PRISMA-ScR) 2018 guidelines [31], a scoping review of the published literature was performed. Searches were conducted in the PubMed and Europe PMC databases using the following free keywords and Medical Subject Headings (MeSH) terms: JAK inhibitor(s), resistance, mutation(s), resistance mechanism, drug resistance, Janus kinases, Janus kinase 2, Janus kinase 1, Janus kinase 3, TYK2 kinase, STAT5 transcription factor, lymphoid leukemia, lymphocytic leukemia, and leukemia. The full details of the search commands and the number of papers retrieved in each search are provided in Supplementary File S1a: Literature Searching. The search, which was restricted to articles published between February 2010 and February 2024, was carried out in February 2024. For the filtering process, we created a Microsoft Excel database that included the following data for each record: PMID or PCMID accession number, title, authors, citation, first author, journal/book, publication year, and DOI (see Supplementary File S1b for a complete list of records). After removing duplicates, a screening was conducted based on titles and abstracts. Articles were selected according to the exclusion criteria. Next, the full texts were reviewed for eligibility based on the inclusion criteria. Finally, we reviewed the citations from the selected articles. Those that aligned with the aim of this review were selected and included if they met the inclusion criteria.

### 3.2. Eligibility Criteria and Study Selection

Articles must have the following characteristics to be included: (1) JAKis resistance must be described using models that recapitulate the genetic or immunophenotype profiles of lymphoid leukemic cells. For preclinical studies, (2) the molecular resistance to JAKis must be supported by experimental data, and the experimental conditions must be specified. For case reports, (3) the JAKis therapeutic regimen specifications must be available, and JAKis clinical resistance must be supported by evidence of a lack of significant clinical improvement not attributable to adverse events or JAKis-related toxicity. Review articles, editorials, and editorial comments were included only if they aligned with the primary objective of this review, and the cited original articles were not already included in the collection of selected articles (see Supplementary File S1c: Screening by Full Text Review and Records from References). From the eligible articles, we selected those focusing on JAK inhibition in Lym-L that contained evidence of resistance to JAKis used in the investigation or currently approved by the FDA for clinical use.

Studies were excluded if they fell into one or more of the following categories: (a) articles on topics unrelated to the focus of this review, (b) articles describing resistance to drugs other than JAKis, or the influence of JAKis or JAK/STAT deregulation on processes unrelated to JAK inhibition in diseases other than leukemia, (c) clinical trials of JAKis or other molecules in diseases other than Lym-L, (d) articles not written in English or without an abstract available, (e) conference materials (see Supplementary File S1d for screening by title and abstract).

The retrieved articles were obtained by two reviewers which worked independently in the screening methodology. Disagreements regarding study inclusion were discussed collectively by all the authors.

### 3.3. Definitions

Based on the literature concepts, we defined molecular resistance as the partial or complete inability of JAKis to eliminate phosphoprotein activity or gene expression profiles related to JAK/STAT signaling hyperactivation. Resistant cells should exhibit cell viability or growth factor independence at increasing concentrations of JAKis [11,15,27,28,30,32,33,34,35,36,37,38]. Clinical resistance is defined as an absence of reduction in disease symptoms that is not attributable to adverse events or toxicity during JAKi’s regimen administration [11,39].

### 3.4. Data Collection, Charting, and Synthesis of Results

The following data from the selected articles were extracted to identify mechanisms of molecular resistance to JAKis in Lym-L.

We collected the following data from preclinical studies: (a) references, (b) the most relevant features of the screening models, (c) JAKis experimental conditions, including the concentrations used for inhibition assays, exposure periods, and whether a control was included, (d) main findings supporting molecular resistance, including measurements associated with JAK inhibition, such as IC_50_ values, proportions of phosphorylated blanks, and changes in gene expression or other phenotypes, and (e) the proposed mechanism of molecular resistance. The data collected from preclinical studies also included JAKi-resistance mutations presented in tables and figures detailing their localization within JAK family protein domains and their molecular interactions associated with resistance.

We provide the three-dimensional localization of the mutations retrieved from the literature, using the JAK crystallographic structures available on the Protein Data Bank and the PyMOL 4.6 software. We also consulted genomic datasets of patients, including COSMIC, PeCAN St. Jude, and the cBioPortal Pediatric Acute Lymphoid Leukemia Phase II Target 2018 dataset, to obtain more information about the possible clinical significance of the JAKi-resistance mutations retrieved from the literature. The mutations observed in patients with Lym-L were recorded and classified according to the AMP/ASCO/CAP 2017 guidelines. The CancerVar database (https://cancervar.wglab.org, accessed on 19 February 2024) was used for the interpretation and reporting of sequence variants [40,41].

The following data were collected from case reports: (a) references, (b) disease classification, (c) initial patient condition before JAKi treatment (e.g., genetic features of leukemic cells, disease stage, and the standard chemotherapy schedule), (d) JAKi-therapeutic regimen dosage and administration time, (e) underlying JAK/STAT abnormalities, (f) JAKi-treatment outcomes, and (g) observations supporting JAKi-clinical resistance.

### 3.5. Critical Appraisal

The Critical Appraisal Skills Program (CASP) checklist was used to review the quality of the research included in the present study. Studies with experimental measurements or clinical observations before and after molecular resistance were considered “highly reliable,” while those that reported this information only for sensitive or resistant isolates were labeled as “less reliable” (see Supplementary File S1e: Quality assessment).

## 4. Results

### 4.1. Selection of Sources of Evidence

**Figure 1:**
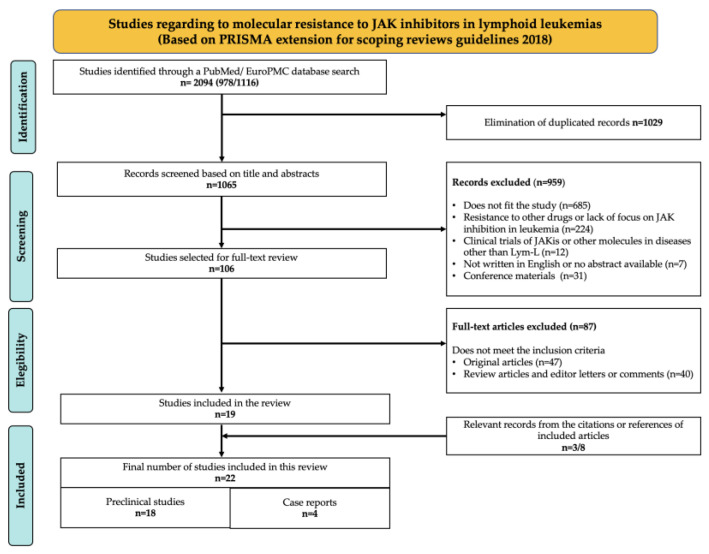
Sequential steps of the research process based on PRISMA-ScR 2018 (Extension for Scoping Reviews).

A total of 2094 articles were initially obtained from the PubMed and EuroPMC databases. After removing duplicate records, 1065 articles were screened based on their titles and abstracts, and 106 were eligible for a full-text review. Of these articles, 19 were selected because they fulfilled the inclusion criteria. Additionally, eight articles that had not been previously considered were retrieved from the citations and references of the selected articles, and three of these were selected due to their compatibility with the review. In total, 22 articles were selected for inclusion and data extraction (Figure 1).

### 4.2. Sources of Evidence

The 22 selected articles were grouped into two main categories:

(1) Preclinical studies with JAKi-resistant models generated in vitro or in vivo, which recapitulate Lym-L features to simulate potential clinical scenarios. This category included a total of 18 articles (see Table S1).
(2) Case reports, which described the outcomes of JAK-mutated Lym-L patients who experienced chemotherapy failure, consented to be treated with a JAKi therapeutic regimen, and developed JAKi clinical resistance. This category included a total of 4 articles (see Table S2).

### 4.3. Critical Appraisal of Sources of Evidence

Of the selected articles, only the case report of Gomez-Arteaga A. et al. (2019) [42] was classified as a less reliable study because it reports features only before the JAKi treatment. The remaining articles fulfilled the criteria for highly reliable studies as they describe the measurements or clinical observations before and after the appearance of JAKi resistance. (see Supplementary File S1e: Quality assessment).

### 4.4. Synthesis of the Results

**Figure 2:**
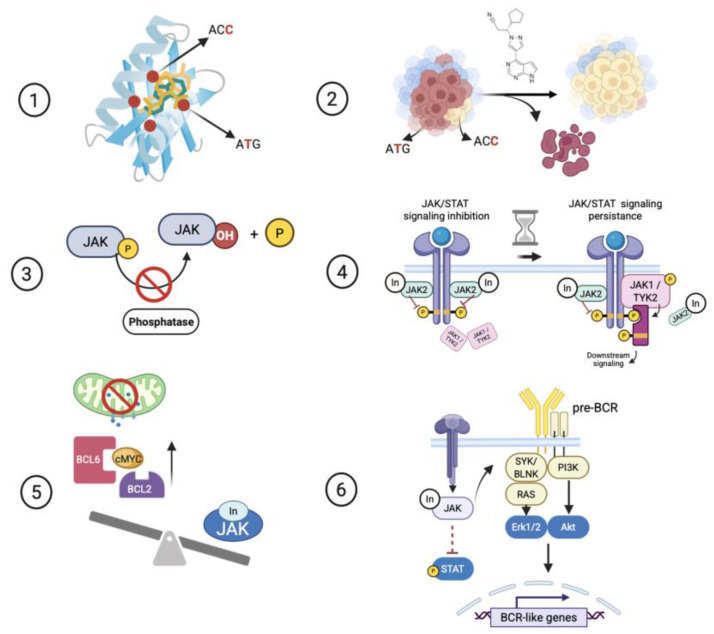
The mechanism of molecular resistance to JAKi in lymphoid leukemias. (**1**) Point mutations in the kinase domain prevent the JAKi binding. (**2**) The cooperative effect of JAK-activating mutations promotes the emergence of a subclone with a higher JAKi threshold. (**3**) Inactivation of phosphatases reduces the response to JAKi. (**4**) Chronic exposure to JAKi promotes the JAK/STAT signaling persistence through the formation of a heterodimeric receptor (**5**) JAK inhibition promotes the overexpression of antiapoptotic survival proteins, which reduces the response to JAKi. (**6**) JAK inhibition promotes the activation of associated pre-BCR signaling pathways and crosstalk signaling, which promote the shifts toward BCR-like identity, defined by partial cell differentiation and survival, upon JAK inhibition. In: Inhibitor.

The evidence from the 22 selected studies allowed us to categorize the molecular resistance to JAKis into two main categories:

Genetic resistance: This is observed when the evasion of JAK/STAT signaling reduction upon JAKi exposure is associated with specific mutations that impair recognition of the inhibitor molecule within drug-binding sites, or with the cooperative effect of JAK-activating mutations that overcome JAK inhibition (Figure 2, Tables S1 and S2) [11,15,24,28,29,30,33,34,35,36,37]. Regarding the JAKi-resistance mutations collected from preclinical studies, these were summarized in Table 1. Additionally, some of these mutations were registered in genomic datasets of patients. Table 2 presents the features and classification of these mutations according to the 2017 AMP/ASCO/CAP guidelines.

Functional resistance: A lack of JAK signaling inhibition is indirectly associated with JAK proteins and is promoted by the inactivation of JAK/STAT signaling negative regulators, such as phosphatases, or by the activation of adaptive gene expression programs or cross-talk signaling pathways induced by acute or chronic exposure to JAKis (Figure 2, Tables S1 and S2) [27,32,37,38,44].

## 5. Discussion

In this section, we describe and discuss the specific molecular mechanisms of JAKi resistance in Lym-L based on the evidence provided by the articles included in this scoping review. However, first, some basic aspects of the normal structure, regulation, and function of JAK proteins are briefly outlined to provide a comprehensive understanding of the JAK malfunction involved in molecular resistance.

### 5.1. JAK Normal Protein Structure, Regulation, and Role in Lymphoid Development

**Figure 3:**
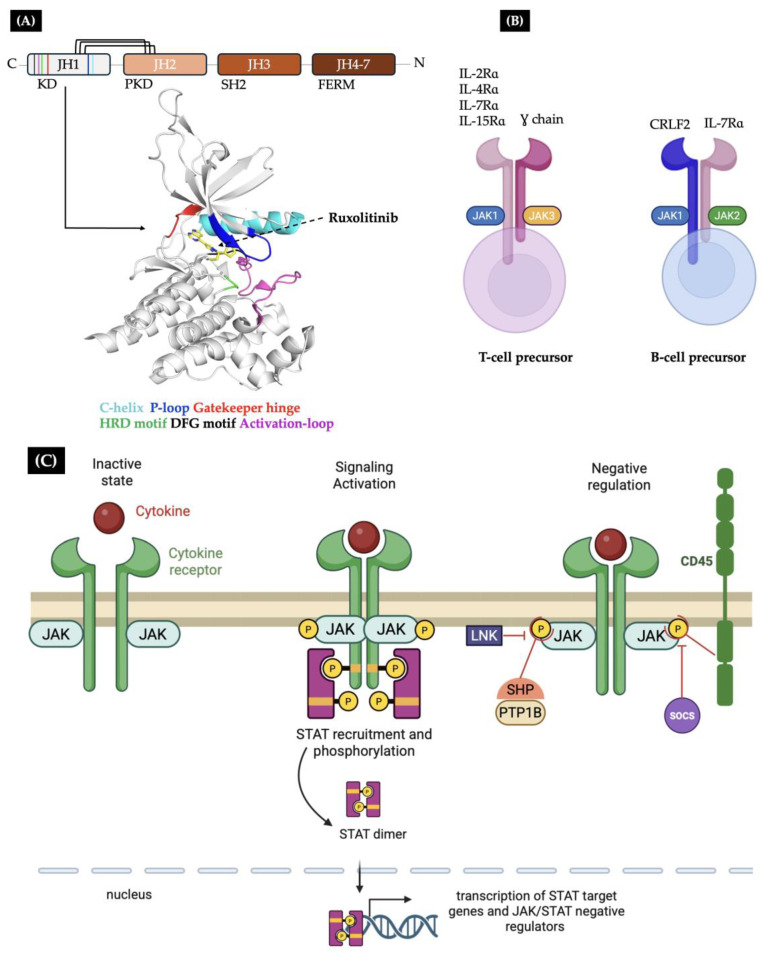
The structure and normal function of JAK proteins. (**A**) The general structure of JAK proteins consists of four functional domains containing JH homology regions. The PKD and KD maintain autoregulatory interactions (represented by black lines). (**B**) The crystallographic structure of the JAK2 kinase domain in complex with ruxolitinib (PDB: 6VGL). Regions involved in catalytic activity are highlighted in different colors. (**B**) Overview of JAK receptor associations in lymphoid lineage cells. (**C**) A summary of the JAK/STAT signaling pathway and its negative regulation. Ligation of a cytokine receptor leads to a transition of the associated JAK molecules from an inactive state (**left**) to an activated state (**middle**). Activated JAKs then phosphorylate tyrosine residues within the intracellular region of the receptor to enable the recruitment and phosphorylation of STATs, which are downstream effectors. There are many layers of negative regulation (**right**), including phosphatase activity (CD45, PTP1B, and SHP1/2) and SH2 domain-containing regulators from the LNK and SOCS families.

The four members of the JAK family share a common and highly conserved domain architecture consisting of seven homology regions (JH1-JH7). The N-terminal region comprises the Src homology 2 (SH2) and four-point-one, ezrin, radixin, and moesin (FERM) domains (JH3-JH7), which mediate binding to the cytoplasmic tails of various cytokine receptors. The JH2 and JH1 regions fold into the pseudo-kinase domain (PKD) and the catalytic tyrosine kinase domain (KD), respectively, at the C-terminus. The PKD lacks the residues necessary for catalytic activity, whereas the KD possesses a β-sheet N-lobe and a gatekeeper hinge region that facilitates ATP/ADP binding and release. Within the ATP-binding site, the KD has a glycine-rich P-loop (GxGxxG) and an HRD motif, which includes the catalytic aspartic acid. The DFG motif is essential for binding magnesium and for ATP orientation (Figure 3A) [2,3,9].

JAK proteins bind to a repertoire of hematopoietic heterodimeric cytokine receptor chains to form a competent cytokine receptor complex. In T-cell precursors, JAK1 associates with cytokine-specific receptor chains (IL-2R, IL-4R, IL-7R, and IL-15R), and JAK3 is anchored to the common γc chain. In B-cell precursors, on the other hand, JAK2 cooperates with JAK1 and associates with the heterodimeric CRLF2-IL7Rα receptor to activate the downstream signaling pathway of thymic stromal-derived lymphopoietin, which is necessary to induce proliferation and differentiation (Figure 3B) [45,46].

The catalytic activity of JAK proteins is controlled by intrinsic events such as the phosphorylation of the conserved tyrosine residues located in the activation loop of the KD. This is associated with the catalytically competent kinase conformation and is distinct from the inactive kinase conformation. Additionally, the PKD is directly adjacent to the KD and, through autoinhibitory molecular interactions, prevents its inappropriate activation in the absence of a stimulus. Extrinsic regulation includes the action of various phosphatases, including SHP1, SHP2, TCPTP, CD45, and SOCS proteins. These phosphatases act directly on JAK itself in a negative feedback loop (Figure 3C) [3,45,46,47].

Specifically, SHP1 is primarily expressed in hematopoietic cells and directly associates with TYK2, JAK1, and JAK2, whereas SHP2 is expressed throughout the body and binds to JAK1 and JAK2. TCPTP (also known as PTPN2) is a T-cell protein tyrosine phosphatase that dephosphorylates JAK1 and JAK3. Unlike the previously mentioned phosphatases, the CD45 tyrosine phosphatase receptor, which is encoded by the PTPRC gene, dephosphorylates phosphotyrosines in the activation loops of all four JAKs. Lastly, SOCS family members induce the degradation of JAK-associated cytokine receptors. This results in receptor turnover (Figure 3C) [2,9].

### 5.2. Point Mutations Around the ATP-Binding Site of the Kinase Domain Confer Genetic Resistance to JAKis

The development of resistance mutations in response to treatment is a well-established mechanism against tyrosine kinase inhibitors in hematological malignancies [47,48]. Based on this, to anticipate the JAKi molecular resistance in a subset of Lym-L patients, random and site-directed mutagenesis approaches were used to identify amino acid residues critical for evading JAKi molecules [11,15,24,28,29,30,33,34,35,36,37]. Most of the randomly mutagenized models possess an initial JAK/STAT-activating lesion, which is typically observed in patients with Lym-L and is responsive to JAKis. However, the acute or long-term exposure to JAKis produces new mutations that prevent JAKi from binding to the ATP-binding pocket or the allosteric site, conferring molecular resistance to multiple type I or II JAKi (Table 1).

Resistant mutations in the gatekeeper hinge region: These mutations were generated in BaF3 cells by deprivation of IL-3 growth factor or by long-term exposure to ruxolitinib [15,24,30,34]. The JAK1 p.F958V and JAK2 p.Y931C mutations involve homologous residues, which are in the hinge region. These mutations display a double effect, confer a strong resistance to type I JAKis, and possess oncogenic activity with a transforming potential in BaF3 cells (IL-3 growth independence) and the ability to promote a constitutive JAK/STAT signaling (Table 1 and Table S1) [15,28,34,49].

**Figure 4:**
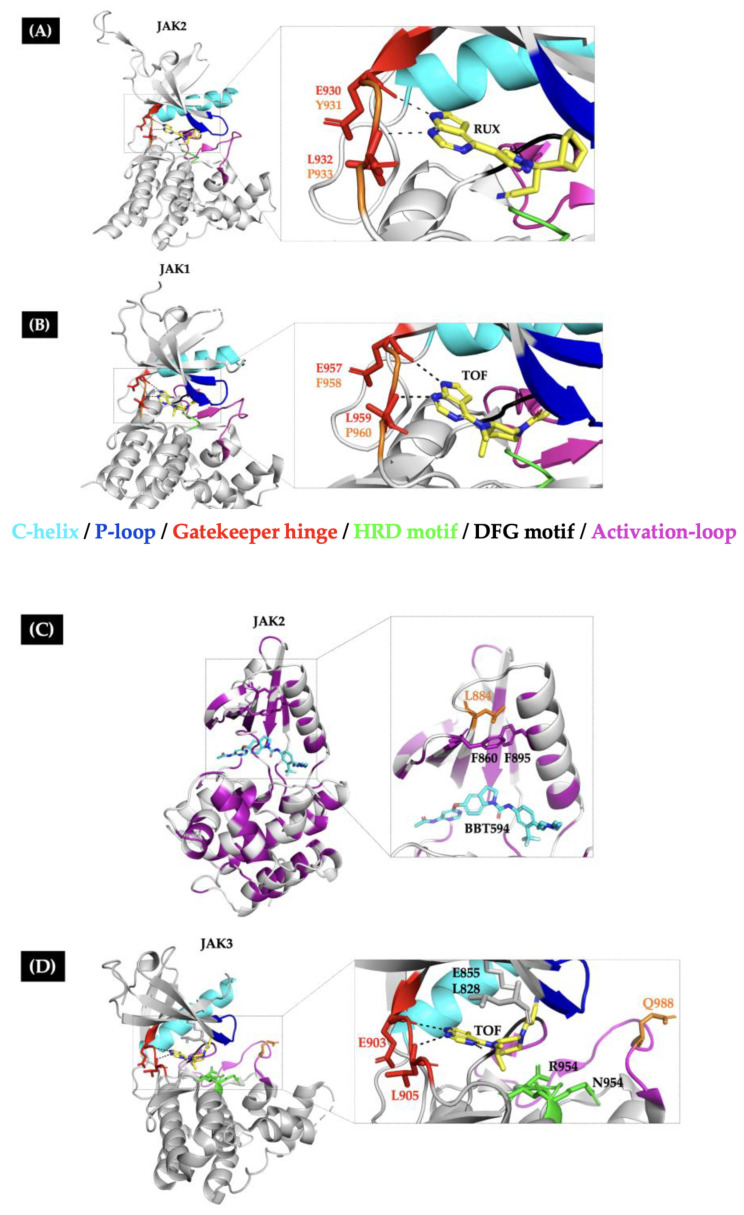
Molecular interactions involved in type I/II JAKis anchoring. The 3D structures of the kinase domain of JAK proteins in complex with inhibitor molecules. (**A**) JAK2 in complex with ruxolitinib (RUX) (PDB:6VLG) (**B**) JAK1 in complex with tofacitinib (TOF) (PDB:3EYG). (**C**) JAK2 in complex with the type II inhibitor BBT594 (PDB:3UGC). The purple residues are hydrophobic; the hydrophobic core, consisting of L884, F895, and F860 residues, allows BBT594 to bind to the allosteric pocket. (**D**) JAK3 in complex with tofacitinib (TOF) (PDB:3LXK). The inhibitor molecule is anchored to the backbone of the hinge residues (red) through hydrogen bonds represented as black dashed lines. Residues affected by JAKi-resistance mutations are marked in orange.

Kesarwani M et al. (2015) performed a molecular docking analysis of the ruxolitinib molecule and the JAK2 crystal structure. They observed that the inhibitor molecules can occupy the ATP-binding pocket in the active conformation (DFG-in), and that anchoring to the ATP-binding cleft requires hydrogen bonds between ruxolitinib and Glu930 and Leu932, which are in the hinge region. Consequently, mutations in this region confer resistance to ruxolitinib by destabilizing the hydrogen bonds necessary for active site anchoring (Figure 4A) [22,49].

The JAK1 p.P960T/S and JAK2 p.P933R mutations involve homologous residues located in the hinge region and confer moderate resistance to type I JAKi [29,48,49] (Table 1). Conversely, Proline 960/933 residues do not come into direct contact with inhibitor molecules. However, they are conserved among JAK family members, which probably introduces rigidity and leads to a controlled movement of the surrounding residues (Figure 4A,B). Substituting these residues with more flexible amino acids could increase the flexibility of the hinge, resulting in less favorable type I JAKi binding [50,51].

Resistant mutations in the allosteric site: Despite type I JAK inhibitors being ineffective against JAK inhibitor-resistant hinge mutations, type II inhibitors (such as CHZ-808 or BBT594) remain effective because they occupy the ATP-binding pocket in the inactive conformation (DFG-out) and an adjacent allosteric pocket between the P-loop and C-helix structures [15,24,30,34]. However, long-term exposure of BaF3 *CRLF2*-positive cells to type II JAKi produces subclones that harbor new JAKi-resistant mutations, such as JAK2 p.L884P. This mutation abrogates the suppressive effects of type II JAKi and confers the ability to proliferate in the absence of cytokines, thereby hyperactivating the JAK/STAT signaling pathway (Table 1).

Analysis of the crystallographic structure of JAK2 KD in complex with the type II JAKi NVP-BBT594 revealed a hydrophobic interaction between the L884 and F860 side chains of the P-loop and the F895 side chain of the C-helix (Figure 4C). This interaction forms a hydrophobic core that stabilizes the P-loop. The loss of this interaction through the L884P mutation would favor a DFG-in conformation, which is available for type I JAKi binding (such as ruxolitinib), but less available for type II JAKi binding [24].

JAK2 p.L884P is homologous to JAK3 p.L857P/H, which is a JAKi-resistant synthetic mutation that confers resistance to ruxolitinib in T-lymphoblast cells. It also exhibits oncogenic properties, such as constitutive JAK/STAT signaling and independence from the Ƴc chain receptor. In addition, the JAK3 p.L857P/H mutation induces B/T lymphoid and myeloid proliferative diseases in vivo. This finding highlights its pathogenicity [23].

Other resistant mutations surround the JAKi-binding site: In addition to the hotspot regions, mutations near the ATP-binding site of JAK proteins were found in multiple randomly mutagenized lymphoid models. These mutations are JAK2 p.E864K/G935R/G993A, which confer resistance to different preclinical and clinical type I JAKis. The G993A mutation is particularly concerning because it confers resistance to a wide variety of type I and II JAKis. Fortunately, there have been no reports of this mutation in Lym-L patients [15,34].

The JAK2 p.R938Q and JAK3 p.Q988P mutations confer a reduced in vitro sensitivity to ruxolitinib. These mutations are located near the ATP-binding site and the activation loop structures. Due to their location, they could induce conformational changes that impair ruxolitinib recognition (Table 1) [29,35]. However, tofacitinib retains its inhibitory capacity for JAK3 p.Q988P, possibly because the mutation is far from the JAK3 residues necessary for tofacitinib binding (Leu 903, Leu905, Leu828, Lys855, Arg953, and Asn954) (Figure 4D). Unfortunately, the sensitivity to tofacitinib or other JAKi was not studied for JAK2 p.R938Q [1,29,35].

#### 5.2.1. Some Preclinically Characterized JAKi-Resistant Mutations Have Been Identified as Somatic Variants in Patients with Lym-L

**Figure 5:**
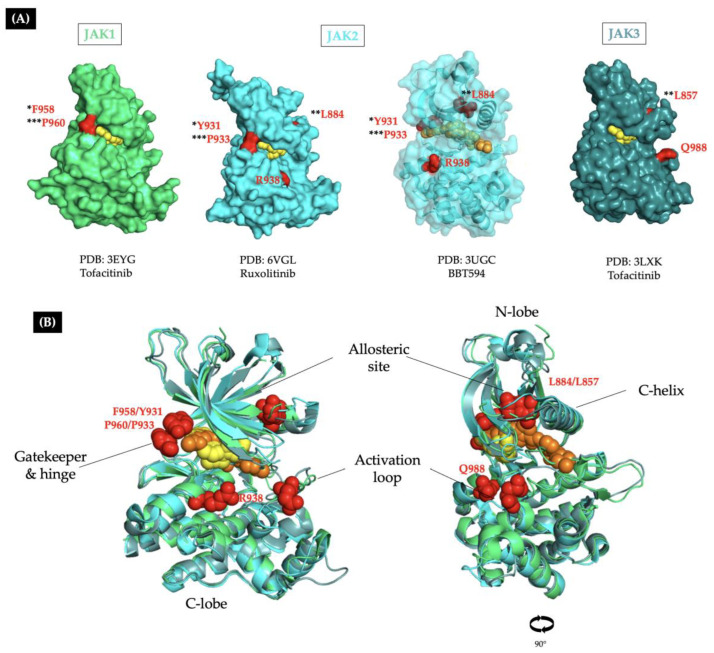
Localization of the preclinically characterized JAKi-resistant mutations observed in patients with lymphoid leukemias. (**A**) 3D structures of JAK protein kinase domains in complex with type I (yellow) or type II (orange) inhibitor molecules. The residues affected by JAKi-resistant mutations reported in patients with Lym-L are marked as red surfaces, and three homologous residues are highlighted with asterisks (*/**/***). (**B**) Superimposition of JAK1-3 crystal structures complexed with type I/II JAKi molecules (PDB:3EYG,3UGC,3LXK). Residues affected by resistance mutations (red spheres) surround the binding pocket. The left figure shows both types of JAK inhibitors and the mutated residues in the hinge and activation loop. The right figure is rotated 90° for a better view of the type-II inhibitor molecule and the mutation in the allosteric site.

According to cancer genomic database searches, some synthetic JAKi-resistant mutations have been reported in patients with Lym-L. These mutations involve hotspot sites located in the gatekeeper, hinge, allosteric pocket, and activation loop regions of JAK proteins (Figure 5 and Table 2).

Hinge JAKis-resistant mutations have been detected in primary leukemia samples obtained from adult T-ALL and pediatric B-ALL patients (Figure 5 and Table 2). The JAK1 p.F958V and JAK2 p.Y931C mutations were recorded as somatic variants with potential clinical significance. In contrast, the JAK1 p.P960S and JAK2 p.P933R were recorded as somatic variants of uncertain clinical significance. However, the Pro960 and the Pro933 residues are homologous to the Phe317/Thr319 hinge residues of ABL1 kinase. These residues are mutated in imatinib-resistant *BCR::ABL1*-positive patients, which reinforces the idea that JAKi-resistant hinge mutations are clinically relevant [52].

Mutations involving the allosteric site of JAK proteins have been detected in patients with Lym-L. The JAK2 p.L884P was annotated as a somatic variant in a patient with B-ALL leukemia positive to *CRLF2* rearrangement. It was classified as an uncertain significant variant. In addition, the JAK3 p.L857P mutation has been identified as a somatic variant with potential clinical significance in adult and pediatric patients with T-ALL (Table 2). Interestingly, the homologous EGFR p.L747P mutation confers resistance to gefitinib in patients with non-small cell lung cancer. This reinforces the suspicion of clinical relevance for the JAK2/3 allosteric JAKi-resistant mutations [24,53]. Both JAK2/3 allosteric site mutations are resistant to a specific type I/II JAKi, but sensitive to others, such as ruxolitinib (for L884P) and tofacitinib (for L857P). This means they could be effectively treated with an appropriate JAKi (Table 1).

Other mutations near the JAKi binding site regions have been detected in patients with Lym-L. Sadras T et al. (2017) described a six-year-old female with B-ALL with a *CRLF2::P2RY8* rearrangement and the JAK2 p.R938Q mutation, which was detected in a JAKi-naive sample obtained at the patient’s diagnosis. The patient was refractory to the AIEOP-BFM-ALL 2009 protocol and subsequently to mitoxantrone and carfilzomib chemotherapy. At relapse, an increase of 9p21 loss of heterozygosity was observed (from 37% to 100%) in CD19+/CD34neg cells, as well as higher levels of pSTAT5, compared with the MHH-CALL4 cell line. This finding suggests cell-intrinsic resistance to ruxolitinib exposure [35]. Genomic database searching revealed another girl with B-ALL harboring the same mutation, which was determined to be a somatic variant of uncertain clinical significance in a relapse sample (Table 2). Unfortunately, it is unclear whether the mutation was present at diagnosis or if the patient had previously been treated with a JAKi regimen. Interestingly, the JAK2 p.R938Q variant was detected in relapse samples from both patients, indicating that the chemotherapy-induced selective pressure had been surpassed.

Lahera A et al. (2024) described a 51-year-old patient diagnosed with T-ALL. The patient was treated according to the ALL/SEHOP-PETHEMA 2011 protocol and received a hematopoietic stem cell transplant (HSCT). Unfortunately, 18 months after HSCT, a relapse of T-ALL was confirmed, and the JAK3 p.Q988P mutation was detected. The mutation was absent at diagnosis, suggesting that it emerged during the progression of the disease. Transfecting the same mutation into BaF3 cells produces a JAK/STAT signaling hyperactivation and reduced sensitivity to ruxolitinib [29]. Searching the genomic database revealed another entry for the same mutation: a 40-year-old patient with T-ALL positive for JAK3 p.Q988P was detected as a somatic variant of uncertain clinical significance in a tumor sample obtained before chemotherapy (Table 2).

#### 5.2.2. The Clinical Relevance of JAKi-Resistance Mutations in Patients with Lym-L

Preclinically characterized JAKi-resistant mutations can be detected as somatic variants in pediatric and adult patients with T-ALL and B-ALL, even in the absence of a prior JAKi therapeutic regimen (Table 2). This may indicate a phenomenon of oncogenic addiction to JAK oncoproteins or deregulation of the JAK/STAT signaling pathway, which can be understood as an exquisite sensitivity of cancer cells to the inhibition of single oncogenes and the development of multiple molecular strategies to bypass inhibition. Oncogenic addiction has been described in other malignancies that have developed resistance mutations in response to tyrosine kinase inhibitor-based therapies [54].

Currently, the robust knowledge about the response to JAKi in hematological malignancies is provided by the experience using ruxolitinib as part of the MPN treatment. Until now, JAKi-resistant mutations have not been identified in these patients, even in cases of ruxolitinib treatment failure. This clinical behavior has been associated with other biological mechanisms of resistance [11,39]. Conversely, laboratory-engineered mutations that confer resistance to JAKi are not identified as recurrent variants in the genomic landscape of JAK-deregulated Lym-L [4,50,55]. However, some patients harbor these mutations as somatic variants of potential or uncertain clinical significance even before chemotherapy initiation, and the mutated clones can support the selective pressure and progress to relapse (Table 2 and Table S2). Based on these facts and preclinical evidence, we cannot rule out the possibility of clinical resistance to a JAKi-based therapeutic regimen in patients with Lym-L.

### 5.3. The Cooperation Between Double JAK Mutants Confers Genetic Resistance to JAKi

Most of the activating JAK protein mutations are in the PKD, disrupting autoregulatory interactions between the PKD and KD and leading to JAK/STAT signaling hyperactivation (Figure 3A) [56]. Springuel L et al. (2014) performed random mutagenesis in growth-factor-independent T-lymphoblast cells that were long-term exposed to ruxolitinib or CMP6 for two to three weeks (Table S1) [37]. These cells harbor an initial activating mutation in the *JAK1* or *JAK3* genes. After exposure to ruxolitinib or CMP6, the cells developed a secondary activating mutation in the corresponding allele (compound heterozygous) or in another gene of the JAK family. The cells exhibited a diminished sensitivity to JAK inhibition, suggesting that JAKi-resistance could be conferred by secondary activating mutations that do not affect cell sensitivity to JAKi when they are present independently [37].

Some patients with T-cell leukemia seem to recapitulate the scenario of cooperative action of activating mutations (Table S2). Wong J et al. (2020) reported an adult patient with relapse of T-ALL who presented with two *JAK3* activating mutations and experienced failure with FLAG chemotherapy. The patient was treated with a tofacitinib-based therapeutic regimen with the addition of dexamethasone. Unfortunately, tofacitinib did not produce a significant clinical response in this patient, either as a single agent or in combination with dexamethasone. In the post-tofacitinib sample, the frequency of the JAK3 p.M511I allele increased from 33% to 93%, and the frequency of the JAK3 p.V657W also increased from 37% to 97% in the T-ALL cells. These similar allele frequency values before and after the JAKi exposure strongly suggest that both mutations coexisted in the same clone [57].

In another case, Greenplate A et al. (2018) reported a 62-year-old female with T-PLL who experienced failure to salvage chemotherapy. She was subsequently treated with a ruxolitinib therapeutic regimen for 116 days due to the presence of activating JAK1 p.V658F and JAK3 p.M511I mutations. Unfortunately, the patient developed ruxolitinib clinical resistance and died of the disease. At relapse, the frequency of the JAK3 p.M511I allele increased from 5% to 28%, whereas the frequency of the JAK1 p.V658F allele had decreased from 40% to 18% in the T-PLL cells. Additionally, the post-ruxolitinib sample exhibited increased JAK/STAT signaling, characterized by higher pSTAT5 levels compared to the pre-ruxolitinib sample. A cell-intrinsic resistance to ruxolitinib and tofacitinib was confirmed “ex vivo” [58].

Springuel L et al. (2014) found in in vitro models that concomitant activating mutations in *JAK1* and *JAK3* appear to cooperate to activate the JAK/STAT pathway by increasing the activity of heterodimeric JAK1/3 receptor complexes. This results in a reduced sensitivity to type I JAKi [37]. However, for the patient in the Greenplate et al. (2018) study, the allele frequencies in the post-ruxolitinib sample suggest that both JAK mutations co-occurred but were not part of the same clone. Hence, the constitution of a heterodimeric JAK1/3 receptor is not possible. Instead, in this patient with more than one JAK-mutated clone, JAKi exposure seems to promote the emergence of a clone with an increased inhibitor concentration threshold. This leads to JAKi molecular resistance and a poor clinical response. This evidence highlights the possibility of JAKi clinical resistance due to cooperation among double JAK mutants (coexisting in different cell populations or as part of the same clone) in patients with T-cell Lym-L (Figure 2).

### 5.4. The Inactivation of JAK-Negative Regulator Phosphatases Confers Functional Resistance to JAKi

Greenplate et al. (2018) reported a patient who revealed two cell-intrinsic mechanisms that account for resistance to ruxolitinib and tofacitinib. (1) Expansion of the mutant *JAK3* clone, and (2) Downregulation of CD45 phosphatase, a negative regulator of JAK/STAT signaling. In this patient, the post-ruxolitinib sample exhibited two new, distinct leukemic populations, as revealed by CyTOF analysis. One resembles the pre-ruxolitinib sample, while a new population emerged with JAKi resistance and presents intermediate and low levels of CD45 expression at the protein and mRNA level. This correlates with the decreased tyrosine phosphatase activity and elevated pSTAT5 levels observed in the relapse cell lysate (Table S2) [58]. Interestingly, Porcu M et al. (2012) reported that T-ALL patients and T-cell lines harboring inactivating mutations in the *PTPRC* gene (which encodes the CD54 phosphatase) and activating mutations in the *JAK1* or *IL7R* genes exhibit an increased JAK/STAT signaling. They also demonstrated in vitro that downregulation of CD45 sensitizes T cells to cytokine stimulation, whereas overexpression of CD45 decreases cytokine-induced JAK/STAT signaling [59].

Another example of JAKi resistance resulting in the inactivation of JAK/STAT negative regulator phosphatases is provided by Kleppe M et al. (2011). They reported that inactivation of the tyrosine phosphatase *PTPN2*, which occurs through deletion of the entire locus, occurs in 6% of T-ALL cases. They also demonstrated in vitro that downregulation of *PTPN2* promotes lymphoid cell transformation mediated by JAK1 hyperactivation. In addition, they demonstrated that downregulation of *PTPN2* expression in BaF3 cells increases the activation status of both wild-type and mutant JAK1 and reduces responsiveness to JAK inhibition (Table S1). This evidence reinforces the idea that patients with a loss of the JAK/STAT-negative regulator phosphatases may respond less well to JAKi. This warrants further studies into the use of JAKi for the treatment of T-cell Lym-L [44].

### 5.5. The Persistence of JAK/STAT Signaling Confers Functional Resistance to JAKi

In MPN preclinical models, the JAK2-dependent cells survive despite chronic JAK2 inhibition through “JAKi persistence”, which involves the reactivation of JAK/STAT signaling through heterodimerization between JAK2 and JAK1 or TYK2, followed by trans-phosphorylation of JAK2. This phenomenon is reversible by withdrawing the JAK2 inhibitor, which leads to re-sensitization to JAK2 inhibition [60]. Tavakoli Shirazi P et al. (2021) reproduced an equal scenario in B-lymphoid cells. They generated a BaF3 model positive for the *MYB::TYK2* fusion oncogene and exposed it to increasing concentrations of cerdulatinib (a JAK/TYK2 inhibitor) for 151 days. The resulting cerdulatinib-resistant cells showed enhanced and persistent JAK/STAT signaling with co-occurring overexpression. This JAK1 overexpression was reversible with a cerdulatinib withdrawal (Table S1) [8].

Additionally, Sanger sequencing revealed that 60% of cerdulatinib-resistant cells harbored a novel mutation in the kinase domain of TYK2 p.R987Q. However, introducing the *MYB::TYK2* p.R987Q mutation into the parental BaF3 cells did not confer resistance to cerdulatinib. Protein modeling analysis revealed that the mutation produces less favorable binding to the ATP pocket, but does not completely inhibit drug binding. Cells with these mutations display similar drug tolerability compared to controls. This suggests that kinase domain mutations may act as an initial, reversible mechanism of JAKi resistance in vitro, but they are not sufficient to confer robust resistance alone. In contrast, withdrawal of cerdulatinib generated cells that were re-sensitized with attenuated levels of pSTAT5 and pSTAT3, as well as decreased levels of pTYK2 and JAK1 expression (but higher than in control naïve inhibitor cells). This indicates that cooperation between JAK1 and TYK2 is the primary JAKi-resistant mechanism in this model of *TYK2*-rearranged B-ALL (Figure 2) [8].

### 5.6. Upregulation of Prosurvival Proteins Confers Functional Resistance to JAKi

Contrary to the *TYK2*-rearrangement B-ALL model of Tavakoli Shirazi P et al. (2021), another study investigating the effect of long-term exposure to ruxolitinib in *CRLF2*-*r/JAK2* mutated B-ALL suggests that adaptation to prolonged JAK2 inhibition is due to c-MYC expression rather than persistent JAK/STAT signaling activation. This highlights that JAKi-resistance may depend on the genetic background [8]. According to several reports, *CRLF2*-r B-ALL exhibits a variable response to type I JAKi, suggesting that inhibiting a single JAK1/2 is an insufficient therapeutic approach [16,24,34]. Based on this, different groups have analyzed the gene expression changes upon JAKi treatment to identify “adaptative pathways” to explain the ineffective nature of JAK inhibition.

Kim SK et al. (2018) performed a comparative analysis of the genome-wide transcriptome in *Crlf2*-r/*Jak2* mutated B-ALL murine cells exposed to acute or chronic *Jak2* inhibition. They discovered significant positive enrichment of the c-MYC gene signature in cells with chronic *Jak2* inhibition (NES: 1.98–2.4; FDR < 0.001). Nevertheless, c-MYC deregulation causes a broad spectrum of cellular effects that are not fully understood [43,61].

The potent effects of dual targeting of JAK2 and c-MYC were further demonstrated in MHH-CALL-4 cells using a combination of ruxolitinib and JQ1, a c-Myc inhibitor. The combination of ruxolitinib and JQ1 provided superior downregulation compared to either agent alone. The efficacy of dual therapy was demonstrated in vivo. Mice that received the combination of JQ1 and ruxolitinib showed significantly lower peripheral WBC counts, a marked reduction in splenomegaly, and a reduced splenic tumor burden compared to mice treated with vehicle or ruxolitinib as a single agent (Table S1) [43].

Interestingly, the modification of gene expression signatures as a compensatory mechanism to overcome JAK inhibition also occurs as a result of acute exposure and is not restricted to type I JAKi. According to Tsuzuki S et al. (2023), human *CRLF2*-r cell lines exposed acutely (24 h) to ruxolitinib or CHZ-868 (a type II JAKi) display upregulation of *BCL6,* which suppresses the expression of *TP53,* its downstream cell-cycle inhibitor p21 (CDKN2A), and proapoptotic molecules such as FAS, TNFRSF10B, BID, BAX, BAK, PUMA, and NOXA, conferring cells a degree of resistance to JAKi therapy. Interestingly, BCL6 suppression by the FX1 inhibitor reduced the growth of *CRLF2*-r B-ALL cell lines. It also restored *TP53* expression, which was diminished by ruxolitinib, and restored the activity of proapoptotic proteins. In addition, the combination of FX1 and ruxolitinib potently inhibited the growth of leukemia cells. This synergistic effect was absent in non-*CRLF2*-r B-ALL cell lines, suggesting a BCL6 dependency, particularly after ruxolitinib treatment. Consistent with this, the JAK2 and BCL6 inhibition with ruxolitinib and FX1 prolonged the survival of xenograft models of *CRLF2*-r cell lines and *CRLF2*-r ALL patient-derived cells beyond that observed in mice treated with ruxolitinib or FX1 monotherapy [32].

The upregulation of anti-apoptotic proteins as a compensatory mechanism to overcome the JAK inhibition has also been reported in T-cell Lym-L models harboring JAK-activating mutations. According to Waibel M et al. (2013), the analysis of the gene expression profile of Eµ-*TEL::JAK2* T-ALL murine cells revealed the overexpression of pro-survival Bcl-2 family genes, similar to the murine *CRLF2*-r B-ALL model of Tsuzuki S et al. (2023) [32]. The combination of Bcl-2/Bcl-xl inhibitor ABT-737 and the type I JAKi TG101209 mediated prolonged disease regression and cure in mice bearing primary human and mouse *JAK2* B-ALL and T-ALL mutant tumors. Moreover, the combined targeting of JAK2 and Bcl-2/Bcl-xL was able to circumvent and overcome acquired resistance to single-agent JAK2 inhibitor treatment (Table S1) [33].

As with B/T-ALL models, analysis of BH3-profiling (a functional assay that determines a cell’s dependence on different antiapoptotic proteins) in 24 primary samples from patients with T-PLL harboring *JAK1/3* activating mutations revealed a relatively low priming for apoptosis and high dependency on the BCL-2 pro-survival protein. Targeting the JAK/STAT pathway with ruxolitinib increased dependence on BCL-2, leading to enhanced sensitivity to venetoclax (a BCL2 inhibitor) (Table S1). Based on these results, Herbaux C et al. (2021) treated an 80-year-old male patient with refractory *JAK3*-mutated T-PLL with a combination of venetoclax and ruxolitinib, observing a deep response and stabilization of the nonmutated disease. These results suggest that the combination of ruxolitinib and venetoclax is promising for treating T-PLL [21].

### 5.7. The Adaptation Through a Shift Towards Pre-BCR Cellular Identity Confers Functional Resistance to JAKi

As previously mentioned, different authors argue that JAKi is insufficient as a monotherapy against JAK-deregulation [16,24,34]. Hurtz C et al. (2020) demonstrated in Ph-like ALL xenograft models that ruxolitinib does not increase apoptosis or markedly decrease pSTAT5, which returns to transitory baseline levels during treatment. However, in line with other reports, exposure to ruxolitinib not only promotes *BCL6* overexpression in *CRLF2*-rearranged Ph-like B-ALL cells but also enriches the gene expression signature associated with B-cell differentiation and pre-BCR signaling (Table S1). Regarding this, exposure to ruxolitinib or CHZ-868 clearly shifts gene expression toward a pre-BCR cellular identity, characterized by an increase in three main signaling pathways: JAK/STAT, PI3K-Akt-mTOR, and BCR-like (BLNK-ERK). Consistent with this kinase-altered phenotype, the multiple TKI therapeutic approach with ruxolitinib, idelalisib (a PI3K inhibitor), and dexamethasone demonstrates efficacy in signaling inhibition in Ph-like B-ALL human cell lines, and xenograft models [27].

Interestingly, the phenotypic shift toward a BCR-like phenotype in response to ruxolitinib treatment was reported in a 41-year-old male with a myeloproliferative neoplasm and *BCR::JAK2* gene fusion. The patient underwent lymphoid-blast transformation after receiving ruxolitinib as an experimental therapy due to the presence of the *JAK2* gene fusion and the initial chemotherapy failure (Table S2). In this patient, relapse was documented after the third cycle of ruxolitinib, and bone marrow analysis revealed a B-lymphoid blast transformation with typical Ph-like lesions, such as *IKZF1* deletion, and increased JAK/STAT signaling and Pre-BCR signaling gene expression. These features were absent in the sample analyzed before ruxolitinib treatment, which reinforces the preclinical evidence provided by Hurtz C et al., 2020, about the relationship between JAKi treatment and the induction of a pre-BCR phenotypic shift [27,62].

Additionally, the reduction in JAKi sensitivity through crosstalk signaling can be associated with deregulation of RAS signaling. Sasaki K et al. (2022) performed a genome-wide CRISPR-Cas9 screen in Ph-like cell lines exposed to ruxolitinib, which differed in their RAS mutational status (Table S1). They observed that ruxolitinib exposure promoted RAS signaling activation, RAS negative regulator gene enrichment, and the absence of growth-suppressive effects in Ph-like B-ALL RAS-mutated cells. Furthermore, the genetic depletion of the *CRKL* gene (a RAS-MAPK negative regulator) or pharmacological inhibition with gerlitinib enhances the ruxolitinib sensitivity. The antileukemic effects of gerlitinib/ruxolitinib combined therapy were also observed in patient-derived Ph-like B-ALL xenograft models, which showed a better outcome compared with models treated with monotherapy. These results highlight the key role of RAS pathway activation in the survival and sensitivity to ruxolitinib [38].

### 5.8. Strategies to Overcome the JAKi-Resistance in Patients with Lym-L

Based on the evidence summarized in this scoping review, some of the biological resistance mechanisms to JAKi seen in preclinical studies may manifest in clinical scenarios, and they may coexist. However, promising approaches exist to prevent clinical resistance.

**Figure 6:**
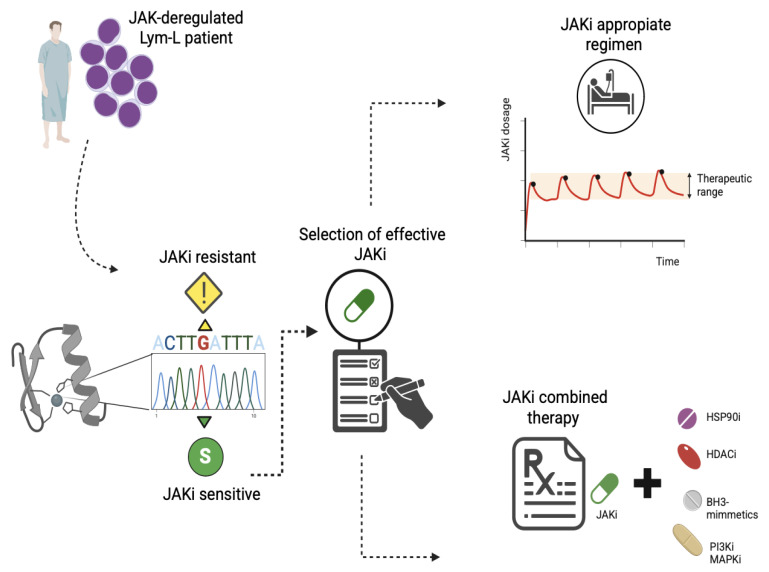
Proposed strategies to overcome the JAKi resistance in patients with lymphoid leukemias. Patients with JAK-mutated lymphoid leukemia should be analyzed for JAKi-resistance mutations. Sequencing of the exons that encode the hotspot regions will allow the identification of mutations that confer resistance to one JAKi but are sensitive to another. This will enable the accurate selection of an effective JAKi. This should be administered intermittently to avoid JAKi resistance by functional mechanisms. Alternatively, JAKis can be administered as a combined therapy with inhibitor molecules that overcome the JAKi-functional resistance mechanisms. Lym-L: Lymphoid leukemia patient. HSP90i: Heat shock protein 90 inhibitors. HDACi: Histone deacetylase inhibitors. PI3Ki: Phosphatidyl-inositol 3 kinase inhibitors. MAPKi: Mitogen-activated protein kinase inhibitors.

Selection of the effective JAKi and dosage regimen: Mutations in the KD of the JAK proteins can result in resistance or sensitivity to different inhibitor molecules. Most mutations are ruxolitinib-resistant, but remain sensitive to other FDA-approved type-I inhibitors, such as tofacitinib or fedratinib (Table 1). The resistance mutations can be detected before the previous use of JAKi, and they are clustered in the gatekeeper hinge, activation loop, and allosteric regions. Therefore, before a JAKi-based therapeutic schedule, the sequencing of the exons encoding the kinase domain will be useful for identifying specific mutations that allow the accurate application of the effective JAKi (Figure 6).

Additionally, the use of type II JAKi, which target kinases in their inactive conformation, is a promising therapeutic approach for overcoming resistance to conventional type I molecules. Available data support the future translation of these inhibitors into clinical trials for the treatment of JAK-deregulated leukemias. According to Wu C et al. (2015) [24], the type-II JAKi CHZ868 potently suppressed the growth of *CRLF2*-rearranged B-ALL cells in mice with human or murine B-ALL. The combination of CHZ868 and dexamethasone synergistically induced apoptosis in *JAK2*-dependent B-ALL cells and improved survival in vivo without increased signs of hematological toxicity. Type II inhibitors may be ineffective against mutations located in the allosteric site, such as the L884P (Figure 4C). However, Arwood ML et al. (2023) discovered and characterized two new type II inhibitor molecules that have the potential to overcome acquired resistance to type II inhibitors in JAK-deregulated B-ALL models. One of these new molecules showed pharmacodynamic activity in vivo [30].

The JAKi regimen can determine the effectiveness of the inhibitory response. Gisslinger H et al. (2014) described two patients with MPN who initially responded to ruxolitinib but later developed resistance. These patients then regained a response after temporary withdrawal from ruxolitinib treatment [63]. This suggests that, for some patients, the JAKi withdrawal may lead to an enhanced therapeutic response. In accordance with this, Tavakoli Shirazi P et al. (2021) demonstrate that JAKi withdrawal could restore the sensitivity to JAK inhibition in a preclinical model of *TYK2* deregulated B-ALL [8]. Additionally, Shank K et al. (2019) demonstrated that a high-dose intermittent schedule was more efficacious than continuous dosing in vivo, without additional toxicity. These findings suggest that intermittent JAKi dosing strategies should be explored in patients with JAK-deregulated Lym-L (Figure 6) [64].

The use of combined therapies: There are currently different approaches based on the combined use of JAKi and molecules that target chaperone proteins, histone deacetylases, pro-survival proteins, and protein kinases, which act as mediators of the JAKi functional resistance (Figure 6).

- HSP90 inhibition: The HSP90 ATPase is a molecular chaperone necessary for the conformational maturation of normal and mutated forms of JAK proteins. Thus, HSP90 is a promising therapeutic target against JAK-deregulated cancers. There are clinical trials with promising results in patients with advanced hematological malignancies [65]. In this context, Weigert O et al. (2012) demonstrated that the HSP90 inhibitor NVP_AUY922 promotes the degradation of both wild-type and mutant JAK2, improves survival in murine models of human and mice *CRLF2*-rearranged B-ALL, and reduces the proliferation of BaF3 *CRLF2*-positive cells harboring kinase domain point mutations. These observations identify the HSP90 inhibitors as promising therapeutic agents for overcoming resistance to JAKi [34].
- HDAC inhibitors: Histone deacetylases (HDACs) play an essential role in regulating HSP90 activity and the transcriptional activity of STATs. Thus, HDAC inhibitors are an attractive target for treating deregulated JAK in Lym-L, overcoming JAKi resistance. Consistent with this, Tavakoli Shirazi P et al. (2021) demonstrated that HDAC inhibitors, such as vorinostat or panobinostat, were effective against *TYK2*-rearranged B-ALL cells that had acquired cerdulatinib (a TYK2 inhibitor) resistance, demonstrating their potential therapeutic use [8].
- Inhibition of antiapoptotic proteins: Based on the evidence provided from preclinical models of B or T cell precursor ALL and two patients with refractory T-PLL (Table S1), JAKi enhances the dependency on antiapoptotic proteins in JAK-deregulated Lym-L cells (Figure 2). Consequently, combined therapy with JAKi and BH3-mimetics, such as FX1, ABT-737, or venetoclax, can overcome resistance to JAK inhibition [21].
- Inhibition of crosstalk signaling pathways: Studies by Hurtz C. et al. (2020) and Sasaki K et al. (2022) highlight the inefficacy of JAKi as a monotherapy. These investigations emphasize the concept of combined TKI therapy against multiple crosstalk kinases, including PI3K, BLNK, and RAS-MAPK, to overcome the JAKi resistance in *CRLF2*-rearranged B-ALL [27,38].

## 6. Strengths and Limitations

To our knowledge, this is the first scoping review providing an updated summary of JAKi resistance evidence described in preclinical models and patients with Lym-L. At the preclinical level, this is a well-documented study highlighting the potential clinical relevance of JAKi resistance molecular mechanisms, including laboratory-engineered resistance mutations, which are present in the baseline composition of the Lym-L molecular background. Additionally, based on the available evidence, some strategies to prevent possible clinical resistance are provided. Nevertheless, an important limitation of this study is the poor availability of information focused of Lym-L patients treated with a JAKi-based therapeutic regimen, since it is under study. Although, considering the possibility of JAKi translation into clinical practice, the discussed points in this review could be useful for patients who will receive JAKi therapy.

## 7. Conclusions

Based on the evidence summarized in this review, different mechanisms of molecular resistance to JAKi can be observed in preclinical models and in patients with Lym-L, and they can play an important role in treatment failure and survival. Additionally, preclinically characterized JAKi-resistant mutations can be detected in pediatric and adult patients with JAK-deregulated Lym-L as somatic pathogenic or uncertain significance variants. It is important to consider that these resistant mutations can be detected in some patients before treatment. Screening for these variants must be performed before the use of JAKis.

Considering the ongoing JAKi clinical trials for Lym-L treatment, we cannot discard the possibility of JAKi resistance in these patients because of the genetic or functional mechanisms summarized and discussed in this review. To effectively use JAKi as therapeutic molecules, we must implement strategies to overcome the JAKi resistance in patients with JAK-deregulated Lym-L.

## Figures and Tables

**Table 1:** Summary of kinase domain mutations conferring molecular resistance to JAKi in Lym-L models.

Ref.	JAK/STAT Initial Activating Lesion	Resistance Mutation	Tested JAKi	IC_50_ Control (µM)	IC_50_ JAK Mutated (µM)	Molecular Mechanism of Resistance
**N lobe and P-loop regions**						
[36]	*TEL::JAK2*	JAK2 p.G831R JAK2 p.E864K JAK2 p.V881A	JAK inhibitor 1	0.25 <0.01 <0.01	10 ^R^ >0.1 ^R^ >0.1 ^R^	Non reported.
[34]	JAK2 p.R683G	JAK2 p.E864K	BVB808 BSK805 JAK inhibitor 1 Ruxolitinib ^C^ Tofacitinib ^C^ TG101348	0.09 3.1 0.19 0.08 0.48 0.60	0.42 ^R^ 8.70 ^R^ 4.67 ^R^ 0.08 ^S^ 0.50 ^S^ 0.81 ^S^	The mutation produces the occlusion of the ATP/JAKi pocket by destabilizing of the P-loop due to a steric clash induced by the charge of the new amino acid side chain.
Allosteric site						
[24]	JAK2 p.R683G	JAK2 p.L884P	BBT594 ^II^ and CHZ-868 ^II^ Ruxolitinib ^C^ Fedratinib ^C^	<0.5 <0.5 <1.0	> 0.5 ^R^ <0.5 ^S^ <1.0 ^S^	This mutation disrupts the interaction between the Phe895 and Phe860, leading to the destabilization of the BBT594 binding pocket.
[23]	JAK3 p.Y100A	JAK3 p.L857P	Ruxolitinib Tofacitinib NIBR3049	1.26 0.17 3.42	2.33 ^R^ 0.32 ^S^ 1.15 ^S^	The mutated residue is homologous to JAK2 L884P and mediates the active/inactive conformational change in the KD.
**Gatekeeper, hinge and activation loop regions**						
[36]	*TEL::JAK2*	JAK2 p.N909K	JAK inhibitor 1	0.25	>10	The mutation produces a steric clash that may push the neighboring V911 residue into the inhibitor binding pocket.
		JAK2 p.Y918H	JAK inhibitor 1	0.25	>10	Not reported
		JAK2 p.M929I	JAK inhibitor 1	0.25	>10	The side chain of M929 may facilitate the proper positioning of JAKi. The M929I mutation is homologous to the ABL1p.T315I resistance mutation.
[15,34]	JAK2 p.R683G *ATF7IP::JAK2*	JAK2 p.Y931C JAK2 p.Y931C	BVB808 BSK805 JAK inhibitor 1 Ruxolitinib ^C^ Tofacitinib ^C^ TG101348 Ruxolitinib ^C^ BMS-911543 AZD-1480 Fedratinib ^C^	0.09 3.1 0.19 0.08 0.48 0.59 0.43 0.66 0.51 0.83	0.42 ^R^ 8.70 ^R^ 4.67 ^R^ 0.38 ^R^ 5.24 ^R^ 1.1 ^R^ 1.98 ^R^ >5.0 ^R^ 3.45 ^R^ 1.77 ^R^	This mutation causes the occlusion of the ATP/JAKi pocket by destabilizing the P-loop due to a steric clash induced by the charge of the new aminoacidic side chain.
[43]	JAK2 p.R683G	JAK2 p.P933R	Ruxolitinib ^C^	IC_50_ non reported	IC_50_ non reported (pJAK2 levels higher than control)	The P933 residue is adjacent to the ATP-binding site and is thought to impart the rigidity necessary for the inhibitor to anchor to the hinge region.
[34,36]	*TEL::JAK2* JAK2 p.R683G	JAK2 p.G935R JAK2 p.G935R	JAK inhibitor 1 JAK inhibitor 1 BVB808 BSK805 TG101348 Ruxolitinib ^C^ Tofacitinib ^C^	0.25 0.19 0.09 0.31 0.60 0.08 0.48	>10 ^R^ 0.51 ^R^ 0.46 ^R^ 8.72 ^R^ 1.29 ^R^ 0.29 ^R^ 0.44 ^S^	The mutation introduces a large, positively charged side chain that may hinder drug binding due to steric hindrance.
[35]	*CRLF2::P2RY8*	JAK2 p.R938Q	Ruxolitinib ^C^	IC_50_ non reported	IC_50_ non reported (pJAK2 levels higher than control)	This mutation may result in allosteric changes within the ATP-binding pocket.
[28]	None	JAK1 p.F958V/C/S/L JAK1 p.P960S/T	CMP6 Ruxolitinib ^C^	0.1 <0.1	>1 ^R^ ≥0.1 ^R^	The mutations abolish the molecular interactions between the hinge region and the JAKi through changes in aminoacidic side chain orientation.
[36]	*TEL::JAK2*	JAK2 p.R975G	JAK inhibitor 1	0.25	>10 ^R^	Replacing Arg 975 with Gly increases the flexibility of the main chain, which may promote opening of the JAKi pocket.
[15]	*ATF7IP::JAK2*	JAK2 p.R983F	Ruxolitinib ^C^ BMS-911543 AZD-1480 Fedratinib ^C^	0.43 0.66 0.51 0.83	>2.0 ^R^ >5.0 ^R^ 3.86 ^R^ 0.71 ^S^	The mutation abolishes the ruxolitinib binding, as the bulkier Phe residue would sterically hinder interaction.
[29]	None	JAK3 p.Q988P	Ruxolitinib ^C^ Tofacitinib ^C^	<1.0 <1.0	>1.0 ^R^ <1.0	Not reported
[15]	*ATF7IP::JAK2*	JAK2 p.G993A	Ruxolitinib ^C^ BMS-911543 AZD-1480 Fedratinib ^C^ CHZ-868	0.43 0.66 0.51 0.83 <2.0	>2.0 ^R^ >5.0 ^R^ 2.83 ^R^ 1.71 ^R^ >5.0 ^R^	A steric clash would occur between the methyl group of Ala933 and the nitrile group of ruxolitinib, which would destabilize the binding of the benzimidazole ring of CHZ-868.
[30]	JAK2 p.R683G	JAK2 p.G993A	Ruxolitinib ^C^ Fedratinib ^C^ CHZ-868 ^II^ BBT-594 ^II^ MFH-6-7-1 ^II^ YLIU-5-162 ^II^	0.37 0.60 0.21 0.17 0.24 0.26	2.30 ^R^ 1.19 ^R^ 2.30 ^R^ 0.27 ^R^ 0.22 ^S^ 0.24 ^S^	
[36]	TEL::JAK2	JAK2 p.P1057S JAK2 p.R1127K	JAK inhibitor 1	0.25	>10 ^R^	Not reported

^R^: Resistant. ^S^: Sensible. ^C^: Clinically used (FDA approved) JAKi. KD: Kinase domain. ^II^ Type II JAK inhibitor molecules.

**Table 2:** Summary of preclinical JAKi-resistant mutations annotated in patient genomic databases.

Reference	JAKi Resistance Mutation	CancerVar Classification *	Patients Annotated in Genomic Databases	Sample ID and Features	Mutation Features
**Allosteric site**					
[24]	JAK2 p.L884P	Tier 3 Uncertain clinical significance.	A case of *CRLF2* rearranged B-ALL	SJBALL021004_D2 Non specified	Somatic VAF 0.26
[23]	JAK3 p.L857P	Tier 2 Potential clinical significance.	A child diagnosed with T-ALL	COSS2770751 Tumor sample obtained after chemotherapy	Somatic, heterozygous
			An adult with T-ALL	COSS2321345 Non specified	Non specified
			A pediatric patient with T-ALL	COSS2770784 Sample collected at diagnosis	Non specified
			A pediatric patient with T-ALL	COSS2770828 Non specified	Non specified
			A child with T-ALL	COSS2770874 Tumor sample collected at diagnosis	Non specified
			A child with T-ALL	COSS2770886 Tumor sample collected at diagnosis	Non specified
			A 9-years old white male with T-ALL	COSS2730627 Tumor primary sample	Somatic
			A 12-years old white male with T-ALL	COSS2730644 Tumor primary sample	Somatic
			An 8.53-years old female with T-ALL	COSS2940234 Tumor primary sample	Somatic
			A patient with T-ALL	SJTALL079673_D1 Tumor sample collected at diagnosis	Somatic VAF 50%
			A patient with T-ALL	SJTALL015689_D1 Tumor sample collected at diagnosis	Somatic VAF 49%
			A patient with T-ALL	SJALL016445_D1 Tumor sample collected at diagnosis.	Somatic VAF 32%
			A patient with T-ALL	SJTALL07967_D1 Tumor sample collected at diagnosis.	Somatic VAF 50%
**Gatekeeper, hinge, activation loop and DGF regions**					
[28]	JAK1 p.F958C	Tier 2 Potential clinical significance	An adult with T-cell Lymphoma/Leukemia	COSS2488735 Non specified	Somatic
	JAK1 p.P960S	Tier 3 Uncertain clinical significance	An 8.6 years-female patient with B-ALL iAMP21 positive	SJBALL030072_D1 Primary tumor sample analyzed before treatment	Somatic VAF 0.35
			A 42-year-old male with T-ALL	COSS2629905 Tumor sample	Somatic heterozygous
[15,34]	JAK2 p.Y931C	Tier 2 Potential clinical significance	A 14-year-old female patient with B-ALL	COSS2843904 Primary tumor sample	Somatic
[43]	JAK2 p.P933R	Tier 3 Uncertain clinical significance	An 8.7-years old child with *CRLF2::IGH* positive B-ALL	COSS1715748 Primary tumor sample	Somatic
			An 8.7-years old female patient with B-ALL	COSS1231802 Non specified	Heterozygous somatic mutation
			A case of pediatric B-ALL	SJBALL021439_D2 Diagnosis sample	Somatic mutation VAF 0.25
[35]	JAK2 p.R938Q	Tier 3 Uncertain clinical significance	A female child diagnosed with B-ALL	COSS2873651 Relapse tumor sample	Somatic
[29]	JAK3 p.Q988P	Tier 3 Uncertain clinical significance	A 40-years old male with T-ALL	COSS2513936 Tumor sample collected before chemotherapy	Non specified

* Based on AMP/ASCO/CAP 2017 Guidelines.

## Supplementary Materials

The following supporting information can be downloaded at: https://www.mdpi.com/article/10.3390/ijms26189111/s1. Refs. [8,15,21,23,24,27,28,29,30,32,33,34,35,36,37,38,42,43,44,57,58,62] are cited in Supplementary Materials file.

## Abbreviations

The following abbreviations are used in this manuscript:

ABT-737	A BCL-2/BCL-xL inhibitor
ALL	Acute Lymphoblastic Leukemia
AMP/ASCO/CAP	Association for Molecular Pathology/American Society of Clinical Oncology/College of American Pathologists
ATP	Adenosine Triphosphate
AUY922	An HSP90 inhibitor
B-ALL	B-cell Acute Lymphoblastic Leukemia
BCL2	B-cell Lymphoma 2 (anti-apoptotic protein)
BH3	Bcl-2 Homology Domain 3
BSA	Bovine Serum Albumin
BCR	B-cell Receptor
CD45	Cluster of Differentiation 45
CHZ868	A type-II JAK inhibitor
CMP6	A type-I JAK inhibitor
CRLF2	Cytokine Receptor-Like Factor 2
c-Myc	Cellular Myelocytomatosis oncogene
CyTOF	Cytometry by Time of Flight
DFG	Asp-Phe-Gly motif
DMSO	Dimethyl Sulfoxide
FDR	False Discovery Rate
FDA	Food and Drug Administration U.S.
FERM	Four-point-one, Ezrin, Radixin, Moesin domain
FX1	A BCL6 inhibitor
HSP90	HSP90Heat Shock Protein 90
HDAC	Histone Deacetylase
HSCT	Hematopoietic Stem Cell Transplant
JAK	Janus Kinase
JAKi	Janus Kinase Inhibitor
KD	Kinase Domain
Lym-L	Lymphoid Leukemias
MeSH	Medical Subject Headings
MPN	Myeloproliferative Neoplasms
NES	Normalized Enrichment Score
PDB	Protein Data Bank
PI3K	Phosphoinositide 3-Kinase
PKD	Pseudokinase Domain
PRISMA	Preferred Reporting Items for Systematic Reviews and Meta-Analyses
PTPN2	Protein Tyrosine Phosphatase, Non-Receptor Type 2
PTPRC	Protein Tyrosine Phosphatase, Receptor Type C
RAS-MAPK	Rat Sarcoma–Mitogen-Activated Protein Kinase Pathway
SHP1/2	SHP1/2Src Homology 2 Domain-Containing Phosphatase ½
SH2	Src Homology 2 Domain
SOCS	Suppressor of Cytokine Signaling
STAT	Signal Transducer and Activator of Transcription
T-ALL	T-cell Acute Lymphoblastic Leukemia
T-PLL	T-cell Prolymphocytic Leukemia
TKI	Tyrosine Kinase Inhibitor
TP53	Tumor Protein p53
TYK2	Tyrosine Kinase 2
VAF	Variant Allele Fraction
