# Supplementary material for: The Mechanisms of Resistance to JAK Inhibitors in Lymphoid Leukemias: A Scoping Review of Evidence from Preclinical Models and Case Reports
Source: Int J Mol Sci. 2025 Sep 18;26(18):9111. doi: 10.3390/ijms26189111 (PMC12470771; doi:10.3390/ijms26189111)
Supplement: Supplementary file 1 [file ijms-26-09111-s001.zip › Supplementary Tables S1 and S2.pdf]

The Mechanisms of Resistance to JAK Inhibitors in Lymphoid Leukemias: A Scoping Review of Evidence from Preclinical Models and Case Reports

Daniel Martínez Anaya [1,2], Marian Valladares Coyotecatl [1], Maria del Pilar Navarrete Meneses [1], Sergio Enríquez Flores [3] and Patricia Pérez-Vera [1,*]

Supplementary Tables

Supplementary Table S1. Preclinical studies that evidence the JAKi molecular resistance in lymphoid leukemia models
Supplementary Table S2. Case reports of JAKi treatment failure in patients with lymphoid leukemia.

Supplementary Table S1. Preclinical studies which evidence the JAKi molecular resistance in lymphoid leukemia models

Reference	Lymphoid leukemia screening models	Experimental conditions	Main findings	Type of molecular resistance
Hornakova T. et al (2011)[28]	BaF3 cells expressing the JAK1 F958V, P960S/T or the JAK2 Y931C hinge point mutations.<br><br>Control: BaF3 cells expressing the JAK1 V658F or the JAK2 V617F activating mutations	The cells were exposed to CMP6, Ruxolitinib, or DMSO at 0.001 to 1 μM for 48 hrs.<br><br>For the long-term exposure the cells were cultured with CMP6 at 1 μM for 2 weeks.	The tested JAK1/2 mutations conferred resistance to acute exposure to both JAKi, with higher levels of pJAK2 and pSTAT5 and IC_{50} values 10 to 50 times higher than the control.<br><br>Long-term exposure of JAK1 F958V cells to CMP6 caused resistance, with higher expression of mutated JAK1 cDNA and higher levels of pJAK1/pSTAT5 than the control.	Genetic resistance to type I JAKi by *JAK1/2* hinge point mutations
Sadras T. et al (2017) [35]	Primary leukemic cells positive to *CRLF2::P2RY8* and JAK2p.R938Q obtained from a child with B-ALL at diagnosis and relapse**.**<br><br>Control: MHH-CALL4 cell line positive to *CRLF2::IGH* and JAK2p.I682F	The cells were exposed to Ruxolitinib or DMSO at 1 μM for one hour.	The R938Q mutation confers Ruxolitinib resistance ex vivo. The inhibitor reduces the pSTAT5 levels in control cells, whereas the primary leukemic cells maintain high levels of pSTAT5.	Genetic resistance to Ruxolitinib by *JAK2* point mutation.
Lahera A. et al (2024) [29]	BaF3 IL3 dependent cells, HEK293-T and U4A, expressing the JAK3p.Q988P point mutation.<br><br>Control: Cells expressing the JAK3p.M511I or L857P activating mutations.	The cells were exposed to Ruxolitinib, Tofacitinib or DMSO at 0 to 1 nM for 96 hrs.<br><br><u>Other specifications:</u><br>The cells are endogenous deficient in *IL2RG* and *JAK1*.	Cells expressing the JAK3p.Q988P mutation maintain growth ability in presence of Ruxolitinib and show increased levels of pSTAT5 compared to controls, but they remain sensitive to Tofacitinib.	Genetic resistance to Ruxolitinib by *JAK3* point mutation.

Losdyck E. et al (2015) [23].	HEK293 and BaF3 cells harbor the JAK3p.L857P/H point mutation.<br><br>Control: HEK293 and BaF3 cells.	The cells were exposed to Ruxolitinib, Tofacitinib, NIBR3049 or DMSO at 0 to 3 μM for 96 hrs.<br><br><u>Other specifications:</u><br>The HEK293 cells are endogenous deficient in *JAK*3, IL-9Rα and γ chain. The BaF3 cells were knocked down for JAK1 and γ chain siRNA.	The cells expressing the L857P/H point mutation are 10-fold more resistant to Ruxolitinib compared with controls and proliferate even in endogenous deficient or knockdown conditions.<br><br>The L857P/H mutation resulted in STAT5 activation independent of the IL-9Rα/γ chain complex.<br><br>Cells expressing the JAK3p.L857P/H mutation remain sensitive to Tofacitinib and NIBR3049.	Genetic resistance to Ruxolitinib by *JAK3* by point mutation.
Downes CEJ. et al (2021) [15].	Chronic exposure: BaF3 cells harboring *JAK2::ATF7IP* were exposed to Ruxolitinib long term.<br><br>Acute exposure: BaF3 cells expressing one of three different *JAK2* fusion genes and harboring Y931C, L983F or G993A point mutations were obtained by long-term exposure to Ruxolitinib.<br><br>Control: BaF3 cells expressing *JAK2* activating fusion genes without point mutations.	For chronic exposure, the cells were cultured with a dose escalation (50 nM to 1μM) of Ruxolitinib over 3 months.<br><br>For acute exposure, the cells were cultured with 1 μM of Ruxolitinib, BMS-911543, AZD-1480, CHZ-868 Fedtratinib or DMSO for one hour.	The cells chronically exposed to Ruxolitinib develop resistance independent of the *JAK:: ATF7IP* fusion because of the development of JAKi resistance mutations.<br><br>.<br>Cells harboring point mutations (particularly G993A), which are derived from long-term exposure, are resistant to acute exposure to multiple type I JAKi. These cells have higher LD50 values and pSTAT5 levels than control cells.	Genetic resistance to multiple JAKi by *JAK2* point mutations
Marit MR et al (2012) [36]	BaF3 *TEL::JAK2* positive cells harbor one of the following JAK2 point mutations: G831R, E864K, V881A, N909K, Y918H, M929I, Y931C,	The cells were incubated with different concentrations of JAK inhibitor 1 (0.01 to 10 μM) or DMSO for 48hrs	The tested mutations conferred a JAKi resistance as the expressing cells showed growth ability persistence, JAK/STAT downstream signaling, activation and high IC_{50} values compared with controls.	JAK2 point mutations confer genetic resistance to type I JAKi.

	G935R, R975G, L983F, G993A, P1057S, R1127K.<br><br>Control: BaF3 *TEL::JAK2* positive cells without point mutations.			
Weigert O. et al (2012) [34]	BaF3 JAK2 p.R683G positive cells express one of the following point mutations: JAK2p.E864K, Y931C or G935R.<br><br>Control: BaF3 JAK2 p.R683G-positive cells without point mutations.	The cells were incubated with increasing concentrations (0.1 to 10 μM) of JAK inhibitor 1, Ruxolitinib, Tofacitinib, TG101348, BVB808, BSK805 or DMSO. The incubation period was 48 hrs.	Cells expressing the Y931C mutation showed a 2 to >10-fold resistance to all JAKi.<br><br>Cells expressing the G935R mutation were resistant to all JAKi except for Tofacitinib.<br><br>Cells expressing the E864K mutation were resistant to BVB808, BSK805 and JAK inhibitor 1, but remained sensitive to Ruxolitinib and Tofacitinib.	Genetic resistance to type I JAKi conferred by *JAK2* point mutations.
Wu SC. et al (2015)[24]	BaF3 JAK2 p.R683G-positive cells expressing the JAK2 p.L884P point mutation were generated by chronic exposure to JAKi.<br><br>Control: BaF3 JAK2 p.R683G-positive cells without point mutations	The cells were incubated with BVB808, CHZ868 or DMSO at 3 μM for 48hrs.	Cells expressing the L884P mutation showed IC_{50} values which were four to eleven times higher for JAKi than in controls.	Genetic resistance to type II JAKi is caused by a JAK2 point mutation.

Arwood ML. et al (2023) [30]	BaF3 JAK2 p.R683G-positive cells that express the JAK2p.G993A point mutation<br><br>Control: BaF3 wild type cells.<br>BaF3 JAK2 p.R683G-positive cells without point mutations.	The cells were cultured in 500nM of Ruxolitinib or Fedratinib (control type I JAKi) or CHZ868 or BBT594 (control type II JAKi) or MFH-6-7-1 or YLIU-5-162 (newly synthesized type II JAKi) or DMSO for 48 hrs.	The G993A point mutation confers resistance to the control type I/II JAKi, with IC_{50} values higher than those observed in controls, but the mutation remains sensitive to the newly synthetized type II JAKi.	JAK2 point mutation confers genetic resistance to type I/II JAKi.
Springuel L. et al (2014)[37]	*JAK1/3* double mutant subclones were derived from growth factor-independent T-lymphoblast cells which were exposed to JAKi long-term.<br><br>Control: Growth factor-independent T-lymphoblast cells harboring only the initial *JAK1/3* activating mutation.	The cells were cultured with increasing concentrations (0.01 to 1 μM) of CMP6, Ruxolitinib or DMSO for 48hrs.	*JAK1/JAK3* double mutant subclones exposed to JAKi showed IC_{50} values four to seven times higher than the controls, as well as high levels of pSTAT5.<br><br>Double-mutant JAKi-resistant subclones showed a double *JAK* mutant partner or a double mutation in the same JAK family member (compound heterozygous).	Type I JAKi resistance is achieved through the cooperation of activating mutations.

Kleppe M. el al (2011)[44]	BaF3 cells that are IL-3-independent, positive for *JAK1* activating mutations and electroporated with shRNAs against the Ptpn2 transcript.<br><br>Control: BaF3 wild-type cells electroporated with a control shRNA.	The cells were incubated with increasing concentrations of JAK inhibitor 1 (1 to 10000 nM) or DMSO for 24 hrs.	Reducing the Ptpn2 protein using shRNAs shifted the JAK inhibitor-I dose-response curve from 49.3nM in control cells to 94.8nM in Ptpn2 knockdown cells.	Functional resistance to type I JAKi. This is due to the loss of function of the phosphatase negative regulator.
Tavakoli Shirazi P. et al (2021) [8]	BaF3 cells expressing the *MYB::TYK2* fusion were exposed to JAKi long term.<br><br>Control: JAKi naïve BaF3 cells expressing *MYB::TYK2.*	The cells were gradually exposed to increasing concentrations of 0 to 3 μM of Cerdulatinib or DMSO for 144 to 151 days.	The cells exposed to the drug for a long term exhibited an 8.7-fold increase in IC50 value (739 vs. 6,508nM), higher levels of pSTAT5, a 3.3-fold decrease in Annexin V positivity (21% vs. 69%), lower proliferation capacity (cell cycles: 13 vs. 9) and increased JAK1/GAPDH protein expression (>0.1 vs. <0.1) compared with the control.<br><br>Withdrawal of JAKi resulted in re-sensitization of cells, decreasing the IC_{50} value (6508 nM in resistant cells vs 2603 nM in re-sensitized cells) and attenuated pSTAT5/3 levels compared with resistant and JAKi naïve cells.	The cells exhibited functional resistance to type I JAKi by JAK/STAT signaling persistence.
Kim KS. et al (2018)[43]	Mice were transplanted with Eμ-Crlf2-r/Jak2 R683G mutant cells.	The mice were treated with DMSO or JQ1 (a c-MYC inhibitor) at a dose of 50mg/kg, once per day, via intraperitoneal injection. This was administered alone or combined with	After three days of treatment, mice receiving the combined therapy had a significantly lower peripheral WBC count (<$10x10^9$/L vs >$10x10^9$/L), a marked reduction in splenomegaly, and lower levels of pSTAT5 and c-MYC, compared to mice treated with vehicle or single agent.	Functional resistance to type I JAKi was evident through upregulation of pro-survival proteins.

		Ruxolitinib (90mg/kg), given orally twice per day.		
Tsuzuki S. et al (2023)[32]	*In vitro*<br>*CRLF2*-rearranged cell lines were transfected with an active form of STAT5 or a control vector.<br><br>Control: Five non-*CRLF2*-rearranged cell lines.<br><br>*In vivo*<br>Xenotransplant model of *CRLF2*-rearranged B-ALL cell lines or patient derived cells administrated with JAKi and BCL6 inhibitor.<br><br>Control: Mice administered with only DMSO.	*In vitro*<br>The cell lines were incubated with the JAKi Ruxolitinib (1mM) or CHZ868 (0.3 mM) for 3 to 6 hrs.<br><br><u>Other specifications</u><br>1. The cells exposed to JAKi were transfected with an active form of STAT5 or a control vector.<br>2. *CRLF2*-rearranged cell lines were incubated with Ruxolitinib (1μM), FX1 (a BCL6 inhibitor) (20μM), and/or the vehicle (DMSO) for 24 hrs.<br><br>*In vivo*<br>Mice were treated with Ruxolitinib (100 mg/kg/day) by gastric gavage twice daily or FX1 (30 mg/kg/day) by intraperitoneal injection once daily, or a combination of 5 days on 2 days off for 4 weeks.	*In vitro*<br>Ruxolitinib and CHZ868 promote *BCL6* upregulation in *CRLF2*-rearranged cell lines, apparently through STAT5 inhibition. Since *BCL6* upregulation can be reverted by STAT5 forced expression in *CRLF2* rearranged cell lines, but not in non-*CRLF2* rearranged cell lines.<br><br>Ruxolitinib and FX1 synergistically suppress the growth of *CRLF2*-ALL cells (with synergy scores >10), but not in non-*CRLF2* ALL cell lines (with synergy scores <10) in culture.<br><br>*In vivo*<br>Compared to DMSO or FX1/Ruxolitinib isolated treatment (survival probability of around 60 or 70 days, respectively), the administration of Ruxolitinib and FX1 prolonged survival by more than 80 days after the transplantation.	Functional resistance to JAKi is achieved through the upregulation of pro-survival proteins.
Waibel M. et al (2013) [33]	*In vitro*<br>Murine Eμ *TEL::JAK2* T cells.<br><br>Control: C57BL/6 normal thymocytes.	*In vitro*<br>The T-cells were treated with increasing concentrations (0 to 500nM) of ABT-737 (a BCL2	In vitro<br>The comparative transcript profiling analysis revealed that *TEL::JAK2* promotes the upregulation of Bcl2, Bcl-xL and Bim in T cells with *JAK2* deregulation (which is	Functional resistance to JAKi is achived through the

	In vivo<br>Mice bearing Eμ*TEL::JAK2* leukemia cells<br><br>NSG mice xenotransplanted with human *CRLF2*-rearranged/ JAK2p.R683G/T875N mutant B-ALL cells.	inhibitor), with or without 0.5 to 2.5 μM of the TG101209 (a JAKi) or DMSO for 24 hrs.<br><br>*In vivo*<br>Mice bearing transplanted murine *TEL::JAK2* T-ALL cells were treated with ABT-737 (75mg/kg).<br><br>Human tumor-bearing mice were treated with a combination of TG101209 (100 mg/kg bid) and ABT-737 (25 mg/kg).<br><br>The mice were sacrificed within 8 hrs. after treatment.	absent in controls). The antileukemic activity of JAKi is enhanced by BCL2 inhibitors.<br><br>*In vivo*<br>In mice bearing *TEL::JAK2* cells the ABT-737 treatment substantially reduce tumor cells in the peripheral blood, concomitant with tumor cell apoptosis induction and significant spleen weight reduction.<br><br>Combined treatment in human tumor-bearing mice resulted in delayed tumor progression. At the end of a three-week treatment cycle, tumor burden was significantly reduced compared to single-agent treatment. Furthermore, only mice treated with the combined therapy showed a sustained therapeutic response.	upregulation of pro-survival proteins.
Herbaux C. et al. (2021)[21]	Twenty-four primary leukemic samples obtained from patients with T-PLL harboring *JAK3* activating mutations were analyzed using BH3 profiling.<br><br>Control: Twenty-four primary samples from patients with treatment-naïve chronic lymphocytic leukemia (CLL) were used as a comparator for BH3 profiling.	The T-PLL cells were co-cultured with the stromal cell line NKTert and then were exposed to 0 to 1 μM concentrations of Venetoclax (a BH3-mimetic), Ruxolitinib (a JAK1/2 inhibitor), Belinostat (a HDAC inhibitor), PRN694 (an ITK/RTK kinase inhibitor) or DMSO for 24 hrs.	The T-PLL cells were less primed for apoptosis than CLL cells: The mean cytochrome c release was 55.2% for T-PLL vs 78.7% for CLL.<br><br>Treatment with Belinostat or Ruxolitinib selectively increased BCL2 priming in T-PLL cells: Overall BCL-2 priming with Belinostat, Ruxolitinib and PRN694 was 39.16, 14.78% and 2.7% respectively.<br><br>Treatment with Ruxolitinib, Belinostat or PRN694 combined with Venetoclax reduced the viability by 25.8%, 37.06% and 6.67%, respectively.	Functional resistance to JAKi is achieved through the upregulation of BCL2.

Hurtz C.et al (2020) [27]	*In vitro*<br>Ph-like ALL cell lines.<br>Control: Ph+ ALL cell lines.<br><br>*In vivo*<br>1) Murine Ph-like and non-Ph-like models.<br>2) *CRLF2*-rearranged Ph-like B-ALL PDX models and Ph-like cell lines derived xenograft models (CDX).<br>3) Control: Ph+ B-ALL PDX models.	*In vitro*<br>The Ph-like cells were exposed to 1μM of Ruxolitinib or 500nM CHZ868 (a type II JAKi) for 72 hrs. The control cells were exposed to Imatinib 500nM for 3 days.<br><br>The Ph-like and Ph+ cells were incubated with Ruxolitinib and/or Idelalisib (PI3Kδ inhibitor) at 1μM for nine days.<br><br>*In vivo*<br>The animals were treated with Ruxolitinib 2 mg/kg once daily, Parsaclisib (PI3Kδ inhibitor) 1 mg/kg twice daily, and/or Dexamethasone 1 mg/kg once daily for up to 14–21 days, depending on rate of leukemia progression.	*In vitro*<br>The GSEA revealed an enrichment of B-cell differentiation gene sets (*BCL6, BLNK*, and *RAG1/2)* in Ph-like ALL cell lines exposed to Ruxolitinib or CHZ868 and in Ph+ALL cell lines treated with Imatinib.<br><br>A single exposure to ruxolitinib showed a marked decrease in pSTAT5 initially, but pSTAT5 levels returned to baseline after 72 hrs of continued drug treatment. Ruxolitinib did not produce an increase in apoptosis, and cell proliferation was moderately affected in Ph-like cells compared with Ph+ cells.<br><br>Combined exposure to Ruxolitinib and Idelalisib decreased cell proliferation of Ph-like and Ph+ cells, while single TKIs exposure for nine days induced minimal effects.<br><br>*In vivo*<br>Murine Ph-like models were moderately sensitive to Ruxolitinib monotherapy, with diminished pSTAT5 levels similar to those of PDX/CDX Ph-like and Ph+ models.<br><br>Combined TKI therapy with Ruxolitinib, Parsaclisib and dexamethasone demonstrated potent inhibition of triple-pathway (JAK-STAT/PI3K/BCR-like) signaling in *CRLF2*-rearranged Ph-like ALL PDX models.	Functional resistance to JAKi shifts toward a pre-BCR cellular identity as well as crosstalk signaling activation.
Sasaki K. et al (2022) [38]	*In vitro*	*In vitro*	*In vitro*	Functional resistance to Ruxolitinib through

	Ph-like ALL cell lines MUTZ5, MHH-CALL4 and KOPN49<br><br>*In vivo*<br>An *IGH::CRLF2* PDX model.	The cells were treated with Ruxolitinib (100 to 500 nM), Gilteritinib (500 nM) or DMSO alone or in combination for 10 days.<br><br>In vivo<br><br>The PDX models were treated with Ruxolitinib (60 mg/kg), Gilteritinib (an CRKL inhibitor, 30 mg/kg) as single or combined therapy, and DMSO as negative control, administered intraperitoneally once daily for 14 days.	A genome-wide CRISPR/Cas9 screening revealed that MUTZ5 cells exposed to Ruxolitinib exhibit changes in the abundance of sgRNA related to the RAS pathway compared to cells exposed to DMSO.<br><br>The sgRNAs that were significantly depleted in Ruxolitinib-sensitive cells correspond to the *CRKL* and *RAPGEF1* genes which are negative regulators of RAS-MAPK pathway.<br><br>Ruxolitinib monotherapy suppressed the growth of Ph-like ALL lines except for KOPN49 cells, which harbor the *KRAS* p.Glu13Asp activating mutation.<br><br>Ruxolitinib treatment combined with *CRKL/RAPGEF1* depletion by sgRNAs suppresses MUTZ5 cell proliferation. Similarly, Gilteritinib treatment, either alone or combined with Ruxolitinib, reduced viability of all cell lines tested and induced apoptosis.<br><br>*In vivo*<br>The Gilteritinib/Ruxolitinib combination reduce the spleen weight and the number of infiltrating ALL cells in PDX model mice compared with mice treated with Gilteritinib or Ruxolitinib monotherapy.	deregulation of RAS signaling pathway.
JAKi: JAK inhibitor. DMSO: Dimethyl sulfoxide used as vehicle. LD/IC_{50}: Half lethal dose/Inhibitory concentration. shRNA: short hairpin RNA. HDAC: Histone Deacetylase. PDX: Patient-derived xenograft. CDX: Cell line derived xenograft. GSEA: Gene set enrichment analysis. Ph+: Philadelphia chromosome positive. TKI: Tyrosine kinase inhibitor. sgRNAs: single guide RNAs. hrs: hours.				

**Supplementary Table S2. Case reports of JAKi treatment failure in patients with lymphoid leukemia**					
Reference	Patient	Initial conditions	JAKi therapeutic regimen	JAKi therapy outcome	Characteristics of JAKi molecular resistance.
Chen JA. et al (2021)[62]	A 41-year-old male with MPN positive for *BCR::JAK2* that progressed to Ph-like B-ALL	Treatment with hydroxyurea for 2.5 months was discontinued 14 months later due to disease progression.	Ruxolitinib was administered after the hydroxyurea was discontinued.<br><br>The dosage was 5 mg PO BID in 28-day cycles.	<u>Therapy failure</u><br><br>After the 3rd cycle of Ruxolitinib the patient relapsed, and a bone marrow analysis revealed a lymphoblast transformation with CD10+, CD34-, CD79a+, and MPO- markers.	Functional resistance through shifts toward a pre-BCR cellular identity.<br><br>The post-Ruxolitinib sample revealed an *IKZF1* deletion, which was absent in pre-Ruxolitinib sample, as well as high levels of JAK/STAT and pre-BCR signaling gene expression.
Wong J. et al (2020) [57]	A 69-year-old female with T-LBL/T-ALL positive to *JAK3* activating mutations.	The patient received Hyper CVAD/MTX-CYT therapy at diagnosis. At relapse, she was treated with FLAG chemotherapy which was discontinued due to AEs.	Seventeen days after FLAG, Tofacitinib was administrated at 5 mg daily.<br><br>Twenty-one days after FLAG, 40 mg of oral dexamethasone and 5 mg of Tofacitinib were administered daily.	<u>Therapy failure</u><br><br>At 36-day post-FLAG the Tofacitinib was discontinued as it did not produce significant clinical activity, either as a single agent or combined with dexamethasone.<br><br>The duration of Tofacitinib treatment was sufficient to observe a clinical response, and the patient did not show signs or symptoms of malabsorption.	Genetic resistance results from the cooperative action of JAK-activating mutations.<br><br>Increased of *JAK3* mutated clone<br><br><u>Pre-Tofacitinib sample:</u><br><br>JAK3p.M511I VAF 33%<br>JAK3p.R657W VAF 37%<br><br><u>Post-Tofacitinib sample:</u><br><br>JAK3p.M511I VAF 93%

					JAK3p.R657W VAF 97%
Greenplate A. et al (2018) [58]	A 62-year-old female with T-PLL positive for *JAK1/3* activating mutations.	The patient was initially treated with alemtuzumab, CHOP, romidepsin and pralatrexate.	Ruxolitinib was administered at 20mg PO BID for 116 days.	<u>Initial good response:</u><br>After five days, the WBC count decreased from 142,000 to 85,000/ µL. She did not experience any AEs to Ruxolitinib, and her WBC count was stabilized at 60,000/ µL for 110 days.<br><br><u>Therapy failure:</u> At day 116, the WBC count increased to 116,000/ µL. The patient developed thrombocytopenia, and she died because of the disease.	Double resistance by cooperative activating mutations and decreased levels of inhibitory phosphatases<br><br><u>Pre-Ruxolitinib sample:</u><br><br>Normal levels of *CD45*, *CD127*, and *PTPRC* mRNA.<br><br>JAK1p.V658F VAF: 40%<br>JAK3p.M511I VAF: 5%<br><br>Post-Ruxolitinib sample:<br>The original clone was present. Two new subclones with intermediate and low levels of *CD45*, *CD127*, and *PTPRC* mRNA were detected.<br><br>JAK1p.V658F VAF: 18% and detected only in the original clone.<br><br>JAK3p.M511I VAF: 28% and detected in all clones.

Gomez Arteaga A. et al (2019)[42]	A 62-year-old male with T-PLL and double *JAK3* mutations	At diagnosis the patient was treated with alemtuzumab. He relapsed 16 months later and received Romidepsin and Lenalidomide. Due to AEs, his treatment was interrupted, leading to disease progression.	Tofacitinib therapy was offered at an initial dose of 5 mg PO BID, increasing to 10 mg after 15 days of treatment. Ruxolitinib was added one month later.	<u>Initial good response:</u><br><br>Tofacitinib produced well-tolerated AEs and a clinical response. Adding Ruxolitinib stabilized the disease without significant liver function abnormalities.<br><br><u>Therapy failure:</u><br><br>After ten months of combined therapy, the patient developed lymphocytosis, fever and night sweat. The JAKi were discontinued and alemtuzumab was restarted.<br><br>The patient died due to disease progression.	Genetic resistance is possibly due to the cooperative action of *JAK3* mutations.<br><br><u>Pre-JAKi sample:</u><br><br>JAK3 p.L511I VAF 50%<br>JAK3 p.L875H VAF 10%<br><br>Primary leukemic cells were sensitive to JAKi in vitro<br><br><u>Post-JAKi sample:</u><br><br>Not analyzed<br>(less reliable study).

MPN: Myeloproliferative neoplasm. AEs: Adverse events. PO BID: Administrated orally twice daily. Hyper-CVAD: Combination of Cyclophosphamide, Vincristine, Adriamycin, Dexamethasone. CHOP: Cyclophosphamide, Doxorubicin, Vincristine and Prednisone. MTX: Methotrexate. T-LBL: T cell lymphoblastic lymphoma/leukemia. T-ALL: T cell acute lymphoblastic leukemia. FLAG: Fludarabine, Cytarabine and Filgrastim. VAF: Variant allele fraction.
